# Exploring the stroke burden linked to Kidney dysfunction: trends, predictive insights, and health inequalities

**DOI:** 10.3389/fneur.2025.1673606

**Published:** 2025-11-24

**Authors:** Jiahao Tang, Yuexin Lu, Shunan Shi, Shu Wan, Ming Wang

**Affiliations:** 1 Brain Center, Zhejiang Hospital, Hangzhou, China; 2 Zhejiang Province Engineering Research Center for Precision Medicine in Cerebrovascular Diseases, Hangzhou, China; 3 The Second School of Clinical Medicine, Zhejiang Chinese Medical University, Hangzhou, China

**Keywords:** global burden of disease, kidney dysfunction, stroke, socio-demographic index, inequality

## Abstract

**Background:**

Over the past 30 years, the prevalence of kidney disease has increased, underscoring the need to address the stroke burden linked to kidney dysfunction. This study aims to analyze trends and factors underlying the stroke burden and its subtypes attributable to kidney dysfunction from 1990 to 2021.

**Methods:**

In this study, we used Global Burden of Disease data to assess stroke burden attributable to kidney dysfunction within the GBD framework. Methods included joinpoint models, decomposition analysis, age-period-cohort models, Bayesian models, and health inequality analysis.

**Results:**

The burden of stroke attributable to kidney dysfunction remains significant, with aging as a key factor. By 2021, such strokes caused 676.1×10^3^ (95% CI: 467.78×10^3^, 896.74×10^3^) deaths and 15,009.65×10^3^ (95% CI: 10,939.08×10^3^, 19,133.72×10^3^) DALYs globally. Males experience a higher burden (ASMR of 9.16 and ASDR of 200.83 per 100,000) compared to females (ASMR of 7.17 and ASDR of 150.63 per 100,000). The burden is concentrated in low SDI countries, with trends intensifying for total and ischemic strokes. Projections suggest a decline in overall burden, but an increase in ischemic stroke among younger populations.

**Conclusions:**

The global stroke burden attributable to kidney dysfunction remains significant, with regional disparities. Public health authorities should create targeted guidelines considering economic levels, stroke subtypes, gender, and age to effectively mitigate this burden.

## 1 Introduction

Stroke is the second leading cause of death globally and the third leading cause of combined death and disability (1). From 1990 to 2021, stroke-related deaths and disability-adjusted life years (DALYs) increased by 44% and 32%, respectively, making stroke the largest contributor to neurological DALYs globally in 2021 (2, 3). Despite significant advances in prevention, diagnosis, and treatment, disparities in burden persist between regions with different levels of development (4).

Chronic kidney dysfunction is an increasingly serious global public health concern. In recent years, a large body of evidence has confirmed its strong association with the occurrence of both cerebrovascular and cardiovascular events as well as with adverse outcomes (5–9). Anatomically and functionally, the microvasculature of the kidneys and brain is exposed to similar hemodynamic stress and is vulnerable to damage from factors such as hypertension. In 2009, Ito et al. (10) proposed the “strain vessel hypothesis,” suggesting that short, high-tension vessels branching directly from large arteries are more prone to hypertension-related injury. This mechanism has since been supported by subsequent studies and has been used to explain the pathophysiological link between renal and cerebral vascular injury. Albuminuria, as a marker of glomerular microvascular damage, has also been demonstrated in multiple recent epidemiological studies to be associated with increased risks of both ischemic and hemorrhagic stroke, as well as with worse prognosis and higher disability burden (6, 11, 12). In addition, patients with kidney dysfunction often present with comorbidities such as hypertension, hyperlipidemia, and diabetes. These factors act synergistically to substantially increase the incidence of stroke and exacerbate disability and mortality burdens (11, 13). Clinical studies and systematic reviews consistently show that kidney dysfunction is independently associated with stroke occurrence, post-stroke neurological impairment, recurrence, and mortality (11, 14–16).

However, despite substantial clinical and epidemiological evidence linking kidney dysfunction to stroke onset and outcomes, its global and regional patterns, temporal dynamics, and attributable burden have not been systematically characterized (2). Previous studies have primarily examined patient-level associations or single cohorts, with limited population-based evaluations of disease burden and its temporal evolution.

Based on the Global Burden of Disease (GBD) database, we conducted a systematic analysis of the health burden of stroke attributable to kidney dysfunction within the GBD framework. We further explored the relationship between kidney dysfunction and stroke burden, while predicting future trends in the disease burden. The aim is to provide more theoretical support for the formulation of health policies and to guide stroke prevention strategies for high-risk populations.

## 2 Methods

### 2.1 Overview

This study was based on data from the Global Burden of Disease, Injuries, and Risk Factors Study 2021 (GBD 2021), coordinated by the Institute for Health Metrics and Evaluation (IHME) at the University of Washington, and supported by the Bill & Melinda Gates Foundation. The GBD project aims to provide comprehensive and comparable estimates of health loss due to diseases, injuries, and risk factors across countries and over time, thereby informing global health policy and priority setting. Using a standardized and transparent analytical framework, GBD 2021 estimated the incidence, prevalence, mortality, years of life lost (YLLs), years lived with disability (YLDs), and disability-adjusted life years (DALYs) for 371 diseases and injuries, 288 causes of death, and 88 risk factors across 204 countries and territories from 1990 to 2021.

Detailed descriptions of the data sources, modeling strategies, and validation processes are available in the GBD 2021 methodological reports and related publications (17).

### 2.2 Data source

Data for this study were obtained from the Global Health Data Exchange (GHDx, https://vizhub.healthdata.org/gbd-results/) using the latest GBD 2021 estimates. From the “Risk factor” module, we extracted the mortality and disability-adjusted life years (DALYs) attributable to kidney dysfunction for stroke and its subtypes. DALYs, combining years of life lost due to premature death and years lived with disability, provide a comprehensive measure of disease burden. Stroke burden attributable to kidney dysfunction (1990–2021) was collected across regions, countries, sexes, and Socio-demographic Index (SDI) levels, where SDI (0–1 scale) quantifies regional development based on fertility, education, and income (4). All epidemiological metrics were presented as absolute numbers, age-specific rates, and age-standardized rates.

### 2.3 Definition

According to World Health Organization (WHO) criteria, stroke is defined as a rapid disruption of brain function lasting more than 24 h or leading to death, usually with a focal onset (18). Ischemic stroke is caused by infarction in the brain, spinal cord, or retina, while intracerebral hemorrhage refers to a localized brain bleed not resulting from trauma (19).

Kidney dysfunction is defined as an estimated glomerular filtration rate (eGFR) < 60 mL/min/1.73 m^2^ and/or a urine albumin-to-creatinine ratio (ACR) ≥30 mg/g (20).

The Global Burden of Disease (GBD) 2021 estimated the stroke burden attributable to kidney dysfunction using a comparative risk assessment framework. Population attributable fractions (PAFs) were derived from meta-analyses of systematic reviews and prospective cohort studies and represent the proportion of disease burden that could be prevented if exposure to kidney dysfunction were reduced to the theoretical minimum risk level (17). These PAFs were applied to overall stroke burden—including deaths, disability-adjusted life years (DALYs), and years lived with disability (YLDs)—to estimate the attributable burden, stratified by age, sex, year, and location.

### 2.4 Statistical analysis

#### 2.4.1 Burden description

GBD-derived total stroke, intracerebral hemorrhage, and ischemic stroke burden trends were visualized spatially and temporally. Joinpoint regression (v5.1.0.0; NCI) computed annual average percentage changes (AAPC) in age-standardized rates (ASMR/ASDR) with 95% CIs.

ASMR (Age-Standardized Mortality Rate) and ASDR (Age-Standardized DALY Rate) adjust for differences in population age structure, allowing comparisons across regions and over time. The rates were calculated using the formula (21):


$$\begin{array}{l} ASR=\frac{\sum_{i=1}^{A}a_{i}w_{i}}{\sum_{i=1}^{A}w_{i}}*100,000\; \end{array}$$

(*a*_*i*_: the age-specific rate in the ith age group; *w*_*i*_: the number of people in the corresponding ith age group among the standard population; A: the number of age groups).

The 95% uncertainty intervals (UIs) are generated using Monte Carlo simulations, accounting for both sampling error and non-sampling variance, and report the 2.5th and 97.5th percentiles of the posterior distribution of model estimates (22, 23). In contrast, traditional 95% confidence intervals (CIs) typically reflect uncertainty due only to sampling error.

Global maps illustrated ASMR/ASDR disparities across 204 countries and 21 regions, while SDI-burden correlations delineated development-level associations.

#### 2.4.2 Age-period-cohort and decomposition analysis

To better understand the temporal patterns and underlying drivers of stroke burden, we used an age–period–cohort (APC) model (“Epi” R package, Poisson distribution) to separate overall trends into age, period, and cohort effects (24, 25). The age effect reflects differences in stroke risk across age groups, the period effect captures temporal changes related to healthcare and environmental factors, and the cohort effect indicates variations among birth cohorts due to differences in exposure and lifestyle. We further applied the Das Gupta decomposition to quantify the contributions of demographic, population, and epidemiological changes to stroke burden (26).

#### 2.4.3 Cross-country inequality analysis

Slope (SII) and Concentration (CI) indices measured absolute/relative health inequalities using weighted SDI-linked regression (ASMR/ASDR) and Lorenz curves (4). ower |SII|/|CI| values denote reduced inequality.

#### 2.4.4 Disease prediction

To further analyze changes in disease burden, we constructed a Bayesian Age-Period-Cohort (BAPC) model using R software. This model employs integrated nested Laplace approximations (INLA) for predictive analysis, enabling accurate forecasting of epidemiological trends (21). Based on this model, we projected changes in disease burden across different age groups for the next 15 years.

All statistical analyses were conducted using R version 4.4.1. The reported ASDR and ASMR are per 100,000 people, along with their 95% uncertainty intervals (UI). The SII, CI, and AAPC are presented with their respective 95% confidence intervals (CI).

## 3 Results

### 3.1 Stroke burden attributed to kidney dysfunction

#### 3.1.1 Burden and its trend

In 2021, there were 676.1 × 10^3^ deaths and 15,009.65 × 10^3^ DALYs recorded globally (Additional file 2: Supplementary Table S1). Although the absolute burden has risen, global ASMR and ASDR have continued to decline, with both showing an AAPC of −1.60 (Additional file 1: Supplementary Figure S1; Additional file 2: Supplementary Table S1). Projections suggest this trend will persist over the next 15 years, with ASMR and ASDR reaching 6.99 and 159.23 per 100,000 by 2036 (Additional file 1: Supplementary Figure S2A, Supplementary Figures S3A, D; Additional file 2: Supplementary Table S4).

**Figure 1:**
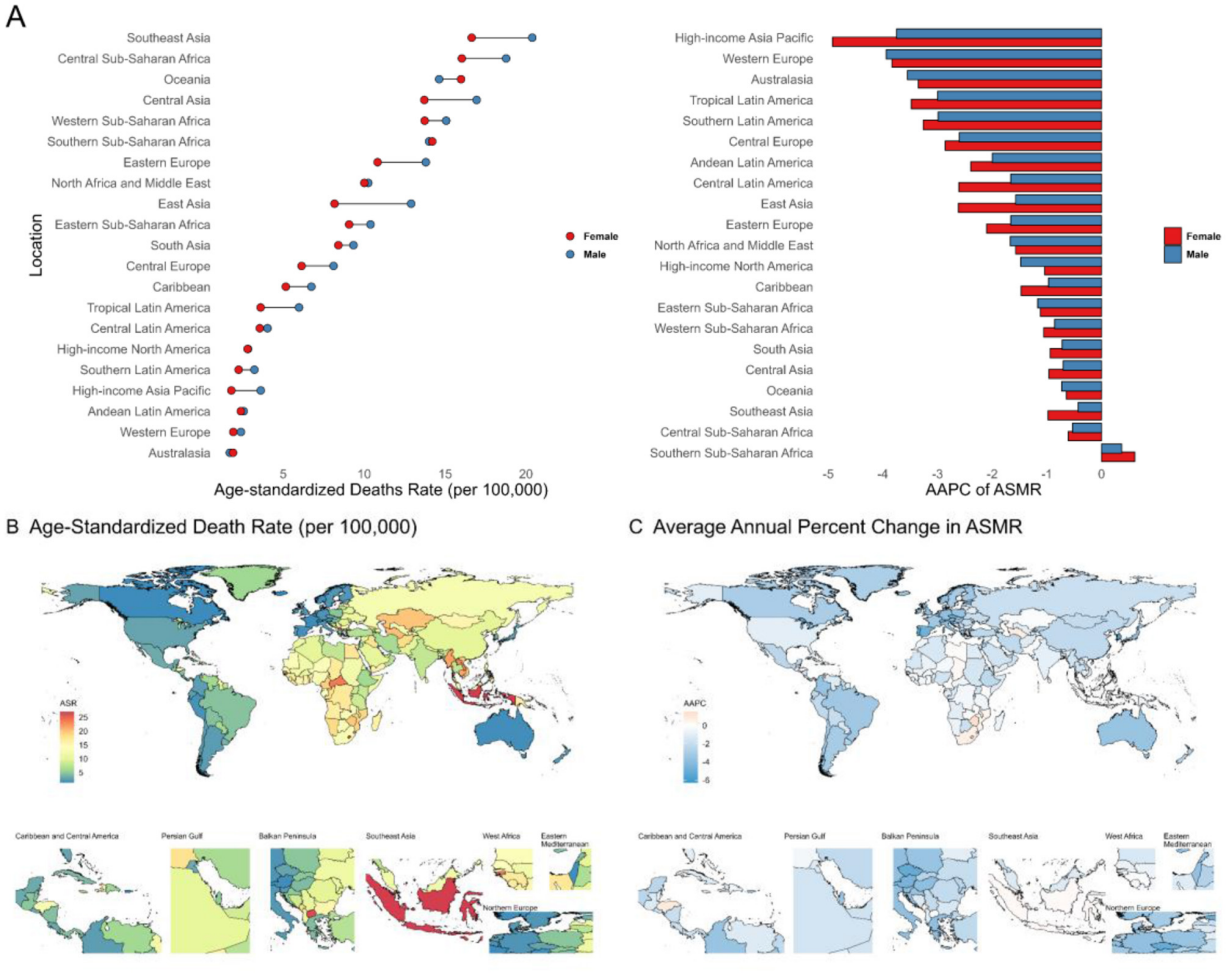
**(A)** Age-standardized death rates and their Average Annual Percent Change from 1990 to 2021in stroke attributable to Kidney Dysfunction in 21 regions by sex. **(B**, **C)** Age-standardized death rates and their Average Annual Percent Change from 1990 to 2021 in stroke attributable to Kidney Dysfunction by country and territory. ASMR, Age-Standardized Mortality Rate; AAPC, Average Annual Percent Change.

Regionally, disease burden is declining across all SDI categories, with high SDI regions consistently reporting the lowest values. As of 2021, low-middle and low SDI regions have surpassed high-middle SDI regions in disease burden (Additional file 1: Supplementary Figure S1; Additional file 2: Supplementary Table S1). Among 21 global regions, Southeast Asia (ASMR: 18.45; ASDR: 415.13), Central Sub-Saharan Africa (ASMR: 17.29; ASDR: 368.58), and Oceania (ASMR: 15.31; ASDR: 347.79) exhibit the highest burden. From 1990 to 2021, all but Southern Sub-Saharan Africa saw a decline, with the fastest reductions in High-income Asia Pacific, Western Europe, and Australasia (Figure 1A; Additional file 1: Supplementary Figures S4A, B, Supplementary Figure S5A; Additional file 2: Supplementary Table S2).

At the national level, low and low-middle SDI countries experience the greatest burden (Additional file 1: Supplementary Figures S4C, D). In 2021, Indonesia (ASMR: 27.06; ASDR: 652.88) and Lesotho (ASMR: 26.76; ASDR: 584.37) had the highest rates (Figure 1B; Additional file 1: Supplementary Figure 5B; Additional file 3: Supplementary Table S1). Most countries showed decreasing trends between 1990 and 2021, led by Singapore (ASMR AAPC: −6.30; ASDR AAPC: −5.50), Korea (ASMR: −5.90; ASDR: −5.80), and Portugal (both ASMR and ASDR: −5.50) (Figure 1C; Additional file 1: Supplementary Figure 5C; Additional file 3: Supplementary Table S1).

#### 3.1.2 Drivers of burden change: aging, epidemiologic change, and population growth

Compared to 1990, the absolute burden of deaths and DALYs in stroke attributable to Kidney Dysfunction increased in 2021. To further explore the underlying causes, we conducted a decomposition analysis. We examined the contributions of three factors—population, epidemiology, and aging—to the age-standardized disease burden. The analysis indicated that population growth contributed to the increase in burden, while epidemiological changes helped reduce it. In high and high-middle SDI regions, epidemiological changes played a major role in reducing the burden. Conversely, in low and low-middle SDI regions, population growth was the dominant factor in increasing the burden. In middle SDI regions, aging, along with population growth, contributed to the rise in disease burden (Additional file 1: Supplementary Figure S6; Additional file 2: Supplementary Tables S5, S6).

#### 3.1.3 Time trends for different age groups and sex

**Figure 2:**
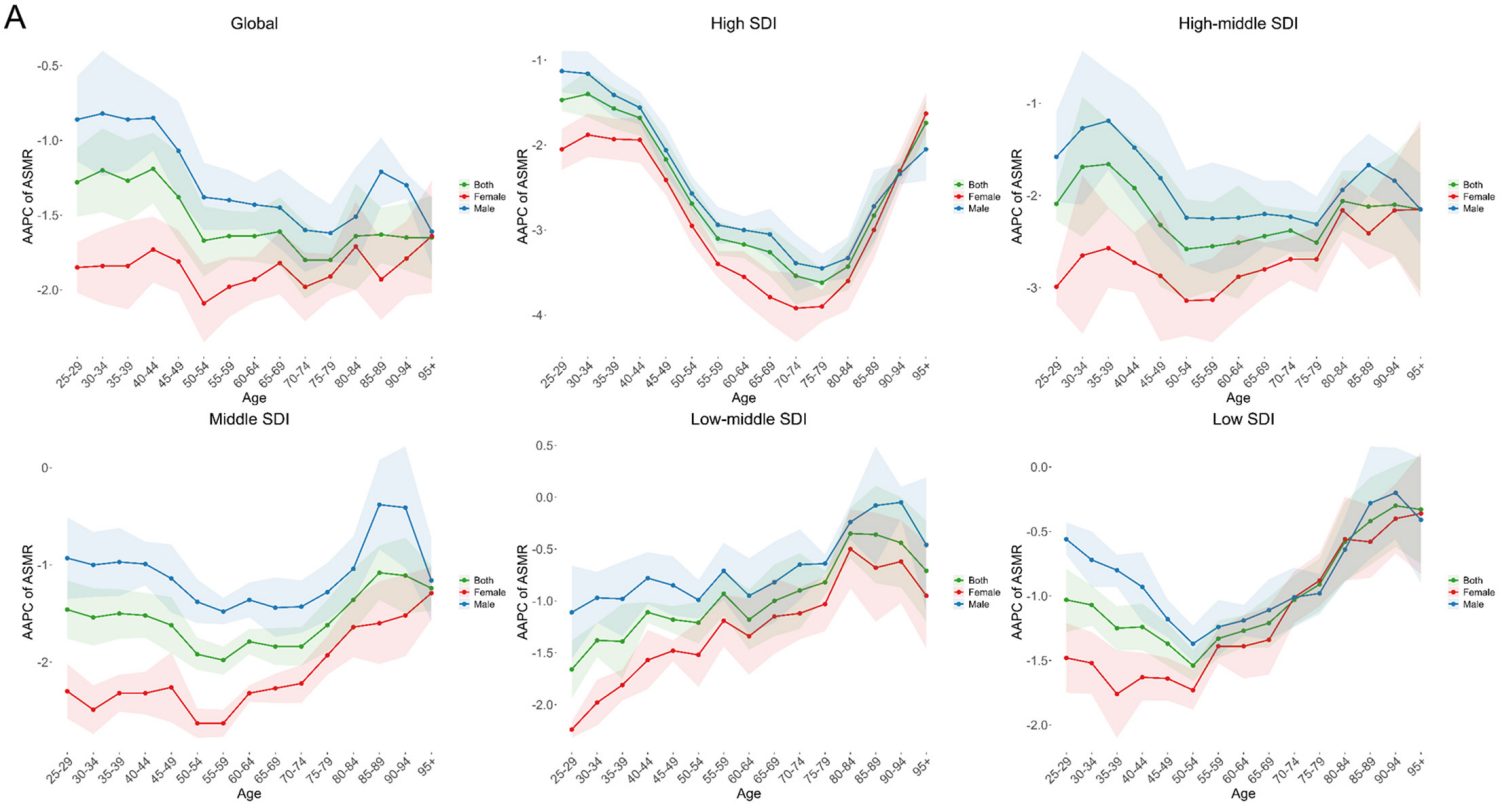
AAPC of age-standardized death rates **(A)** for stroke attributable to Kidney Dysfunction across different age groups by SDI quintiles from 1990 to 2021. SDI, Socio-Demographic Index; AAPC, Average Annual Percent Change.

The AAPC of ASMR and ASDR across different age groups globally and in various SDI regions are all less than 0, indicating a declining trend in disease burden (Figure 2; Additional file 1: Supplementary Figure S7; Additional file 2: Supplementary Table S3). Regions with higher development levels generally exhibit a more significant decline in disease burden. Among different regions, the AAPC for older age groups tends to be smaller, especially in areas with lower development levels. This suggests that the disease burden among the elderly remains an important focus for public health policy.

In terms of sex differences, the ASMR and ASDR in males were 9.16 and 200.83 per 100,000, respectively, in 2021, notably higher than 7.17 and 150.63 in females. Although the overall burden showed a declining trend, the reduction was more pronounced in females (ASMR AAPC: −1.79; ASDR AAPC: −1.87) than in males (both −1.40). This pattern was consistent across age groups, with females showing a faster decline in disease burden than males (Figure 2; Additional file 2: Supplementary Table S1). These findings indicate that males will likely continue to bear a higher burden of stroke attributable to kidney dysfunction and should remain a focus for targeted prevention efforts.

#### 3.1.4 The age, period, and birth cohort effects on stroke attributable to kidney dysfunction

Using an age-period-cohort (APC) model analysis, we examined the impact of individual age, cohort, and period on the disease burden of stroke attributable to kidney dysfunction. Overall, the disease burden increases with age, consistent with the established view that age is a risk factor for disease burden. During the study period, we also observed a declining trend in the effects of period and cohort on the disease burden (Additional file 1: Supplementary Figure S8).

### 3.2 Trends in stroke subtypes burden attributed to kidney dysfunction

#### 3.2.1 Burden and its trends

We assessed the contribution of kidney dysfunction to different stroke subtypes. In 2021, kidney dysfunction was associated with substantial mortality and disability for both ischemic and hemorrhagic stroke. Specifically, it contributed 346.36 × 10^3^ deaths and an ASMR of 4.24 per 100,000 for ischemic stroke, and 329.74 × 10^3^ deaths and an ASMR of 3.86 per 100,000 for hemorrhagic stroke. The corresponding DALYs were 6,956.93 × 10^3^ (ASDR 82.11 per 100,000) for ischemic stroke and 8,052.72 × 10^3^ (ASDR 92.48 per 100,000) for hemorrhagic stroke (Additional file 2: Supplementary Table S1).

**Figure 3:**
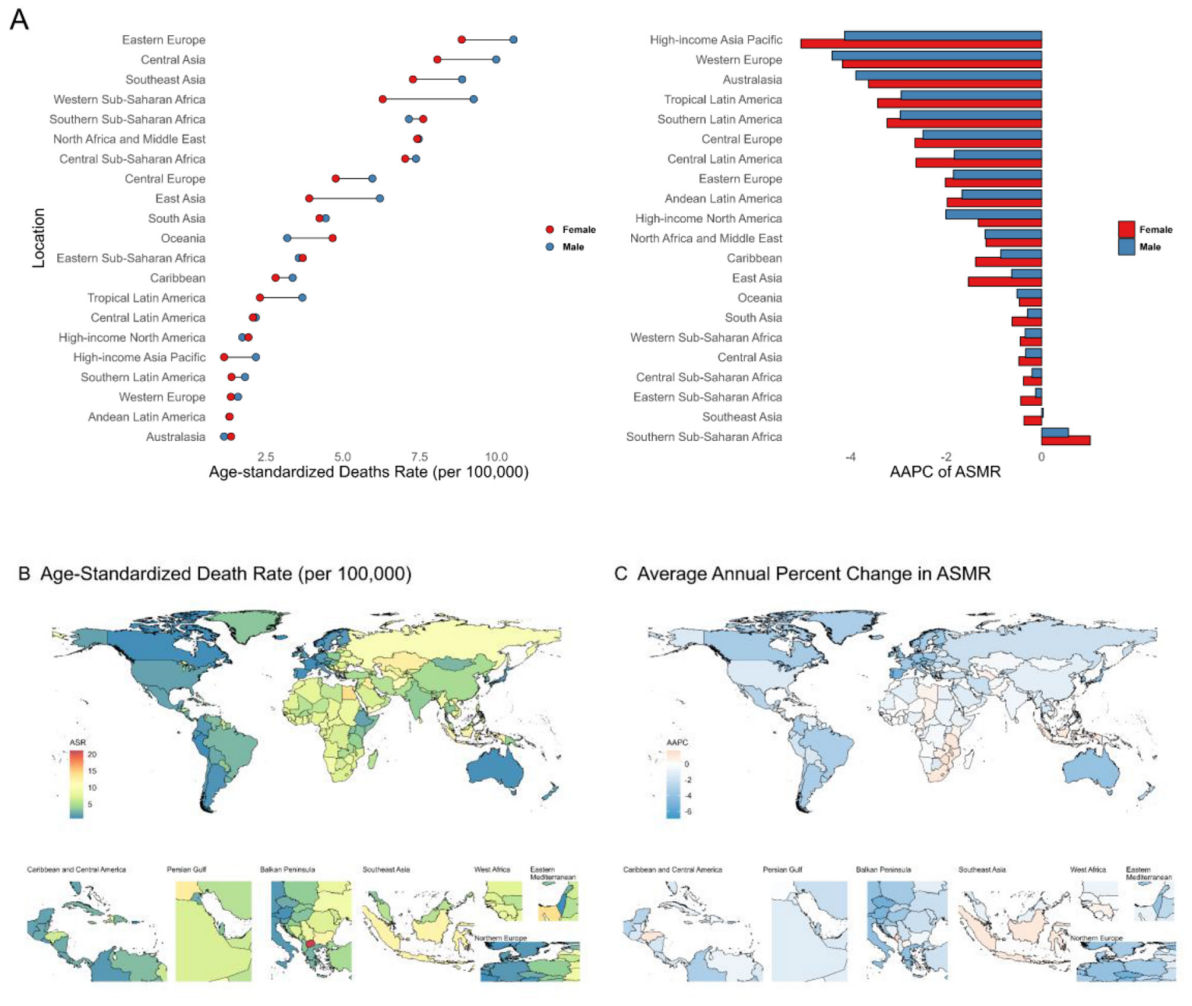
**(A)** Age-standardized death rates and their Average Annual Percent Change from 1990 to 2021 in Ischemic Stroke attributable to Kidney Dysfunction in 21 regions by sex. **(B, C)** Age-standardized death rates and their Average Annual Percent Change from 1990 to 2021 in Ischemic Stroke attributable to Kidney Dysfunction by country and territory. ASMR, Age-Standardized Mortality Rate; AAPC, Average Annual Percent Change.

Since 1990, both ASMR and ASDR of ischemic stroke attributable to kidney dysfunction have declined steadily (Additional file 1: Supplementary Figure S9), with the highest burden now shifting to low-middle SDI regions (Additional file 1: Supplementary Figures S10C, D). Although the global burden is projected to decrease over the next 15 years, an increase among younger populations may occur (Additional file 1: Supplementary Figure S2B; Supplementary Figures S3B, E; Additional file 2: Supplementary Table S4). In 2021, Central Asia and Eastern Europe had the highest ischemic stroke burden (Figure 3; Additional file 1: Supplementary Figure S11; Additional file 2: Supplementary Table S2), with all regions—except Southern Sub-Saharan Africa—experiencing a decline since 1990 (Figure 3; Additional file 1: Supplementary Figures S10A, B). The most significant reductions occurred in High-income Asia Pacific and Western Europe (Figure 3A; Additional file 2: Supplementary Table S2). Nationally, North Macedonia had the highest burden, while Singapore and Portugal showed the fastest declines in ASMR and ASDR, respectively.

**Figure 4:**
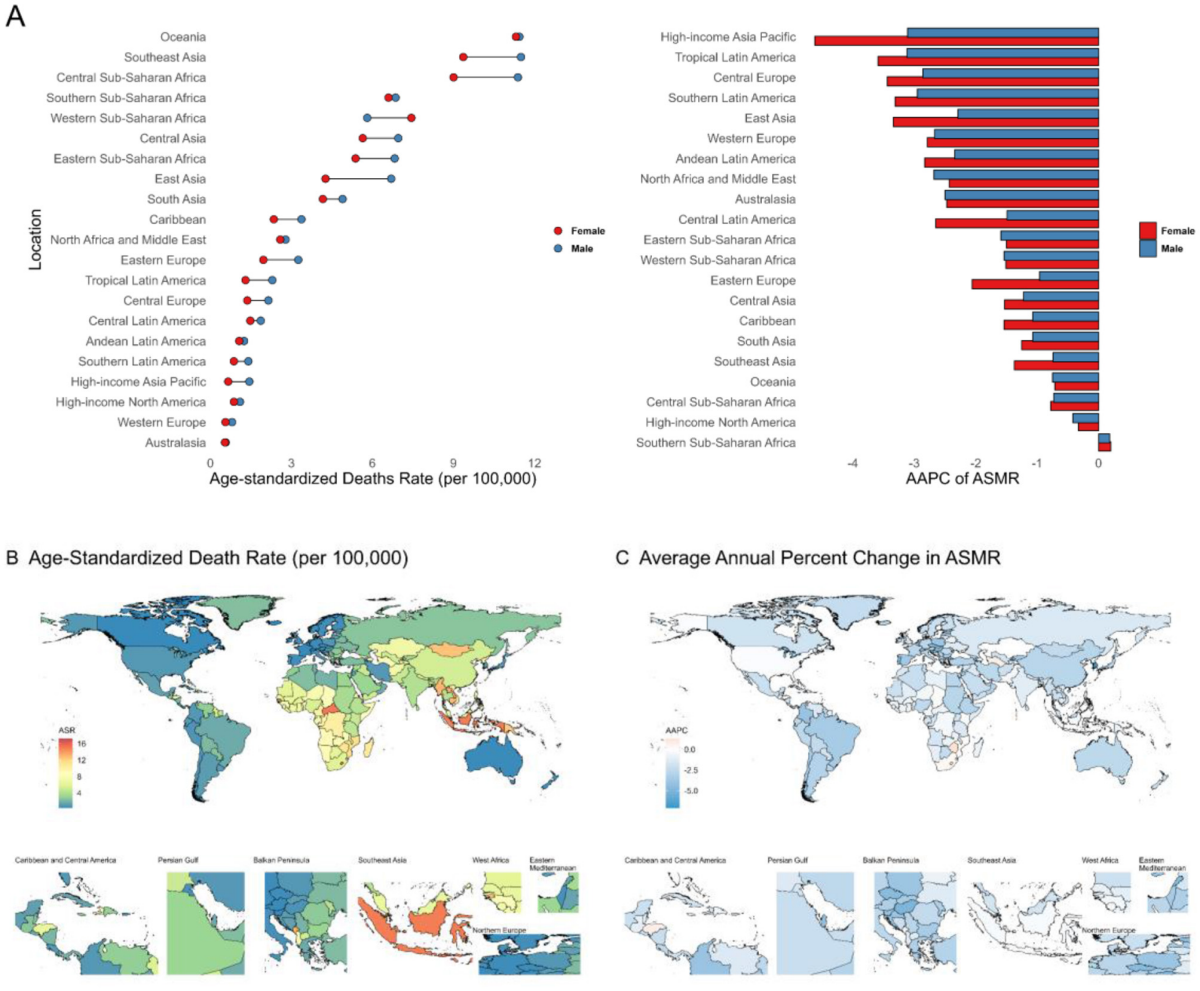
**(A)** Age-standardized death rates and their Average Annual Percent Change from 1990 to 2021 in Intracerebral hemorrhage attributable to Kidney Dysfunction in 21 regions by sex. **(B, C)** Age-standardized death rates and their Average Annual Percent Change from 1990 to 2021 in Intracerebral hemorrhage attributable to Kidney Dysfunction by country and territory. ASMR, Age-Standardized Mortality Rate; AAPC, Average Annual Percent Change.

The global burden of intracerebral hemorrhage attributable to kidney dysfunction has also shown a consistent decline and is expected to continue decreasing over the next 15 years (Additional file 1: Supplementary Figure 2C; Supplementary Figures 3C, F; Supplementary Figure S16; Additional file 2: Supplementary Table S4). From 1990 to 2021, the most significant declines were observed in high SDI regions (AAPC of ASMR −2.31, AAPC of ASDR −2.40) (Additional file 2: Supplementary Table S1). By 2021, the highest burden was concentrated in less-developed regions, particularly Oceania (ASMR 11.37 per 100,000, ASDR 269.54 per 100,000) (Figure 4A; Additional file 1: Supplementary Figure S17, Supplementary Figure S18A; Additional file 2: Supplementary Table S2). The High-income Asia Pacific region showed the steepest decline (ASMR AAPC −3.71; ASDR AAPC −3.62) (Figure 4A; Additional file 1: Supplementary Figure S18A; Additional file 2: Supplementary Table S2). At the national level, Nauru had the highest burden (ASMR 17.23; ASDR 470.05 per 100,000), while Korea had the largest decline (ASMR AAPC −7.10; ASDR AAPC −6.90) (Figures 4B, C; Additional file 1: Supplementary Figures S18B, C; Additional file 3: Supplementary Table S1).

#### 3.2.2 Drivers of burden change: aging, epidemiologic change, and population growth

The results of the decomposition analysis for stroke subtypes are similar to those for overall stroke: population growth has increased the disease burden, while epidemiological changes have reduced it. The main difference between ischemic stroke and intracerebral hemorrhage lies in the role of aging in the burden across different regions. In both subtypes, aging has increased the disease burden in terms of ASDR. However, in the analysis of ASMR, aging has increased the burden for intracerebral hemorrhage, whereas for ischemic stroke, aging has actually reduced the burden, except in high SDI regions (Additional file 1: Supplementary Figures S6, 12; Additional file 2: Supplementary Tables S7–S10).

#### 3.2.3 Time trends for different age groups and sex

Globally, the burden of stroke attributable to kidney dysfunction shows a declining trend across all age groups, though significant differences exist in terms of gender and subtype. For ischemic stroke, the reduction in burden generally exhibits age dependency, with larger declines observed as age increases. In both high SDI and low SDI regions, the decline peaks among middle-aged populations, while changes are less marked in younger and older groups. In low SDI regions, AAPC values for young and elderly males are even greater than 0, indicating an increased burden proportion in these groups (Additional file 1: Supplementary Figures S12, 13; Additional file 2: Supplementary Table S4). Regarding intracerebral hemorrhage, AAPCs peak globally in the 70–74 age group. Younger and older groups show smaller AAPCs, suggesting an increasing proportion of burden in these extreme age groups (Additional file 1: Supplementary Figures S20, 21; Additional file 2: Supplementary Table S4).

Regarding sex differences, the burden of ischemic stroke (IS) and hemorrhagic stroke (HS) attributable to kidney dysfunction was predominantly concentrated in males. In 2021, the ASMR and ASDR for IS were 4.70 and 92.43 per 100,000 in males, higher than 3.84 and 72.89 in females. For HS, the corresponding rates were 4.46 and 108.40 in males vs. 3.33 and 77.74 in females (Additional file 2: Supplementary Table S1).

From 1990 to 2021, females experienced a greater decline in disease burden than males. For IS, the AAPC in ASMR was −1.94 for females and −1.45 for males, while the AAPC in ASDR was −1.68 and −1.24, respectively. For HS, the AAPC in ASMR was −1.79 for females and −1.40 for males, and in ASDR −1.87 and −1.40, respectively (Additional file 2: Supplementary Table S1).

Across all age groups, males overall exhibited higher mortality and DALY rates, whereas females showed a faster decline in burden (Additional file 1: Supplementary Figures S12, 13, Supplementary Figures S20, 21; Additional file 2: Supplementary Table S12). These findings suggest that men remain disproportionately affected by stroke related to kidney dysfunction, highlighting the need for targeted prevention and intervention strategies in this population.

#### 3.2.4 The age, period, and birth cohort effects on stroke subtypes attributable to kidney dysfunction

In the changes of disease burden across different subtypes, an analysis of period effect and cohort effect reveals an overall downward trend during the study period. The role of aging in different subtypes is similar to that in overall stroke, showing a trend of increasing disease burden with aging. However, in the analysis of intracerebral hemorrhage, we found a significant downward trend in disease burden in the 80–84 age group, followed by a gradual increase. This phenomenon is particularly prominent in the analysis of ASDR for hemorrhagic stroke (Additional file 1: Supplementary Figures S15, 22).

#### 3.2.5 SDI-related health inequality in the burden of stroke and its subtypes attributed to kidney dysfunction

Health inequality analysis revealed that the burden of stroke and its subtypes attributable to kidney injury was concentrated in low Socio-demographic Index (SDI) regions. Between 1990 and 2021, the Slope Index of Inequality (SII) indicated widening absolute inequalities in burden across SDI levels for total stroke [ASMR SII: −8.77 (95% CI: −11.36, −6.19) to −11.63 (−13.76, −9.51); ASDR SII: −241.19 (−299.83, −182.56) to −273.79 (−318.35, −229.23)] and ischemic stroke [ASMR SII: −0.35 (−1.69, 0.98) to −4.23 (−5.30, −3.15); ASDR SII: −24.97 (−49.76, −0.19) to −82.50 (−102.73, −62.27)], driven by a greater reduction in high-SDI regions. In contrast, the burden of hemorrhagic stroke remained predominately concentrated in low-SDI areas. However, its SII decreased [ASMR SII: −8.67 (−10.42, −6.93) to −7.53 (−8.60, −6.47); ASDR SII: −219.54 (−260.49, −178.60) to −181.63 (−206.92, −156.34)], indicating reduced absolute inequalities. The Concentration Index (CI) suggested a relative decrease in inequality across all stroke types, potentially attributable to faster population growth in low-SDI regions (Additional file 1: Supplementary Figures S23–25; Additional file 2: Supplementary Table S11).

## 4 Discussion

This study conducted an in-depth assessment of the global stroke burden attributable to kidney dysfunction from 1990 to 2021. Our findings are generally consistent with previous GBD-based analyses, which also reported declining age-standardized rates despite an increase in absolute case numbers, largely reflecting demographic dynamics such as population growth and aging (27, 28). We extend prior research by highlighting substantial regional heterogeneity: the burden remains concentrated in low-SDI countries, particularly in Southeast Asia, Central Sub-Saharan Africa, and Oceania. These disparities may stem from differences in healthcare infrastructure, access to early detection and treatment of chronic kidney disease, and the uneven distribution of stroke prevention programs. Moreover, the slower decline in stroke burden among males, as well as the potential increase in ischemic stroke among younger populations, suggests that current prevention strategies may not be equally effective across all demographic groups. These findings underscore the urgent need for targeted interventions in resource-limited settings to mitigate the future stroke burden attributable to kidney dysfunction.

Clinically, stroke risk is strongly linked to kidney function. Declining glomerular filtration rate (GFR) and elevated urinary albumin are associated with increased stroke risk (29). Patients with end-stage renal disease (ESRD) have significantly higher atrial fibrillation risk compared to the general population (30). Given the rising prevalence of kidney disease, the associated stroke burden warrants concern (20, 31, 32). The mechanisms linking kidney dysfunction to stroke risk are both anatomical and pathophysiological. Anatomically, juxtamedullary nephron arterioles and cerebral penetrating arteries are exposed to high-pressure environments, making them susceptible to hypertensive damage (11). Pathophysiologically, aside from traditional factors (e.g., hypertension, diabetes), non-traditional renal-related risks—such as chronic inflammation, reactive oxygen species, and uremic toxins—promote endothelial dysfunction and vascular injury (11). Impaired kidney function also enhances protein carbamylation, exacerbating dyslipidemia and atherosclerosis risk (33). It contributes to platelet dysfunction and abnormal platelet–vessel interactions, increasing hemorrhagic stroke risk (34). Furthermore, hyperphosphatemia in uremic patients promotes vascular calcification via phenotypic changes in vascular smooth muscle cells (35).

The global stroke burden linked to kidney dysfunction has decreased, mainly thanks to advances in public health and medical care. However, the number of people affected is still increasing, especially in low SDI regions, which may worsen the overall burden (36–39). Aging significantly contributes to this rise. As the global population ages, older adults face higher mortality rates and DALYs compared to younger individuals. Kidney-related diseases in the elderly contribute notably to the global disease burden, as aging leads to reduced kidney function and worsened renal sclerosis (40). This may be due to the reduction in renal cortex volume and increased surface roughness with age, leading to decreased kidney function. At the microstructural level, renal sclerosis characterized by arteriosclerosis worsens with age (41, 42). Aging also accelerates atherosclerosis by impairing vascular mitochondrial function and disrupting mitochondrial autophagy (43). Hypertension and dyslipidemia risks increase with age, leading to poorer outcomes and higher stroke mortality rates in the elderly (44). The observed declines in period and cohort effects since 1990 are likely related to improvements in public health and greater access to healthcare. Enhanced sanitation and infection control have reduced competing causes of morbidity and mortality, allowing more individuals to reach ages at higher risk for stroke and kidney dysfunction (45–47). Meanwhile, expanded healthcare coverage, advances in diagnostic imaging (e.g., CT and MRI), and wider availability of treatments for hypertension, dyslipidemia, and diabetes have improved early detection and management of stroke and its risk factors (48–50). These combined advances have contributed to reductions in age-standardized rates, despite increases in absolute case numbers, particularly in regions with greater medical progress.

From a sex-specific perspective, men bear a greater stroke burden associated with kidney dysfunction, largely due to higher exposure to behavioral risk factors such as smoking and alcohol consumption, as well as the protective effects of estrogen in women (51, 52). Estrogen enhances endothelial function, regulates vascular tone, and reduces oxidative stress and inflammation, thereby mitigating renal microvascular injury and the kidney–brain vascular cascade leading to stroke (53–55). It also delays atherosclerotic progression and stabilizes plaques, supporting its protective role against ischemic stroke (56). In hemorrhagic stroke models, female animals exhibit less cerebral edema, faster inflammatory resolution, and better blood–brain barrier repair, further confirming the neurovascular protective effects of estrogen (57–59). In contrast, men's adverse lifestyle patterns and higher prevalence of hypertension, metabolic syndrome, and insulin resistance exacerbate oxidative damage and vascular dysfunction, increasing stroke susceptibility (17, 60). Sex differences are not fixed attributes of protection or risk but dynamic outcomes shaped by age, hormonal fluctuations, genetic polymorphisms, behavioral and environmental exposures, and disparities in healthcare access. Therefore, sex-specific prevention and intervention strategies should move beyond biological determinants to incorporate lifestyle, sociocultural, and environmental influences that modulate stroke vulnerability.

The number of DALYs and the ASDR for intracerebral hemorrhage are significantly higher than those for ischemic stroke. ICH is recognized as the most destructive and disabling type of stroke, often causing extensive brain injury and associated with complications such as cerebral edema, rebleeding, and hematoma formation (61). Approximately 15%−23% of patients experience hematoma expansion and early neurological deterioration, contributing to poor outcomes (62, 63). Surgical intervention is frequently required to evacuate hematomas, control hemorrhage, and alleviate intracranial pressure, yet these procedures carry substantial risks (64, 65). In contrast, advancements in interventional strategies and stroke management have led to improved prognoses in ischemic stroke patients.

The stroke burden attributable to kidney dysfunction is primarily concentrated in low-SDI countries and regions, likely reflecting disparities in healthcare infrastructure, limited access to early screening and treatment, and insufficient coverage of preventive interventions. Previous studies have shown that evidence-based therapies, diagnostic imaging, and stroke units are less frequently available or utilized in low- and middle-income countries, contributing to delayed diagnosis and poorer clinical outcomes (48, 66, 67). Although age-standardized mortality and DALY rates have declined in these regions, reflecting improvements in stroke detection and management, the absolute number of cases continues to rise, largely driven by population growth and aging (2, 28).

To address global inequalities in stroke burden, targeted policies and interventions are needed. In resource-limited settings, priority strategies include early screening and diagnosis of kidney dysfunction among high-risk populations, strict control of blood pressure and metabolic risk factors, improved access to acute stroke interventions, and capacity building for healthcare providers. Public health education is also critical, with school- and community-based water, sanitation, and personal hygiene programs potentially reducing infection-related adverse stroke outcomes (68–70).

It is important to note that our study is based on the GBD database. In some low-development countries, due to the lack of medical infrastructure and professionals, data may be subject to underreporting and record loss (71). Especially in regions where public health systems are underdeveloped and national conditions are unstable, the available data may not accurately reflect the actual burden of stroke. The GBD study applies standardized modeling approaches (e.g., CODEm, DisMod-MR) and data imputation to address missing data and improve comparability across countries (72). Nevertheless, residual uncertainty may remain, and the estimates for low-SDI regions should be interpreted with caution. Sensitivity analyses specifically addressing the impact of underreporting were not feasible in this study, but this represents an important area for future work to further validate the robustness of regional estimates.

In the context of an increasingly aging population, it is crucial to further explore the impact of kidney dysfunction on stroke and its subtypes, as well as the underlying mechanisms. Our study provides a comprehensive assessment of the global burden and distribution of stroke and its subtypes attributable to kidney dysfunction within the GBD framework. Based on the analysis of disease burden trends, we further explain the underlying cause, which aids in the development of targeted health policy interventions. In addition, we highlight regional inequalities in the global disease burden. This helps to direct more resources and attention to areas in greater need, aiming to optimize global disease care and rationalize resource allocation. Consequently, it improves treatment outcomes and reduces inequalities in the global health burden.

## Data Availability

The original contributions presented in the study are included in the article/Supplementary material, further inquiries can be directed to the corresponding authors.
