# Supplementary material for: Exploring the stroke burden linked to Kidney dysfunction: trends, predictive insights, and health inequalities
Source: Front Neurol. 2025 Nov 24;16:1673606. doi: 10.3389/fneur.2025.1673606 (PMC12683663; doi:10.3389/fneur.2025.1673606)
Supplement: Supplementary file 1 [file Data_Sheet_1.pdf]

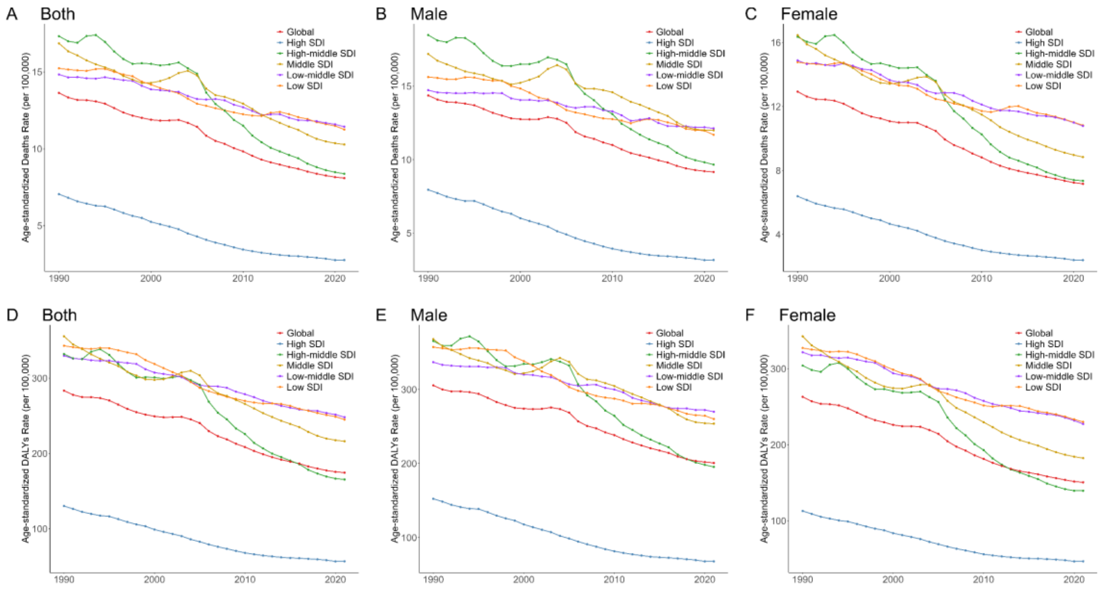

Fig. 1 Age-Standardized Deaths Rate and Age-Standardized DALYs Rate of Stroke Attributable to Kidney Dysfunction by SDI Quintiles for Both Sexes (A and D), Male Population (B and E), and Female Population (C and F), 1990–2021. DALYs Disability-Adjusted Life Years, SDI Socio-Demographic Index.

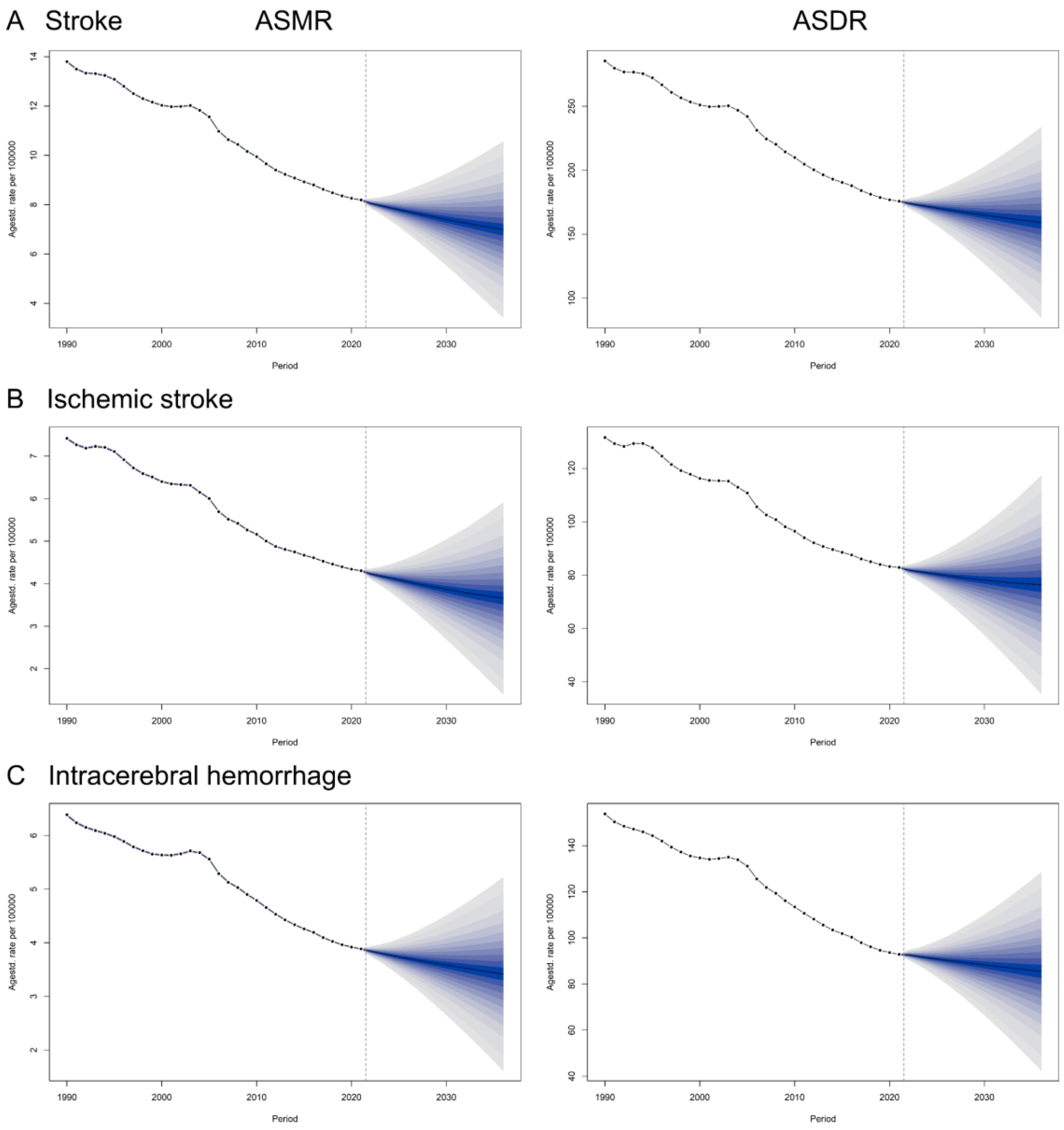

Fig. 2 Trends of age-standardized death rate and DALYs rate of stroke attributable to Kidney Dysfunction from 1990 to 2021 and the forecasts until 2036 globally. The left side presents data on death attributable to Kidney Dysfunction in Stroke (A), Ischemic stroke (B), and Intracerebral hemorrhage (C), respectively. The left side presents data on DALYs attributable to Kidney Dysfunction in Stroke (A), Ischemic stroke (B), and Intracerebral hemorrhage (C), respectively. DALYs stand for Disability-Adjusted Life Years, ASMR Age-Standardized Mortality Rate, ASDR Age-Standardized Disability-Adjusted Life Year Rate.

A

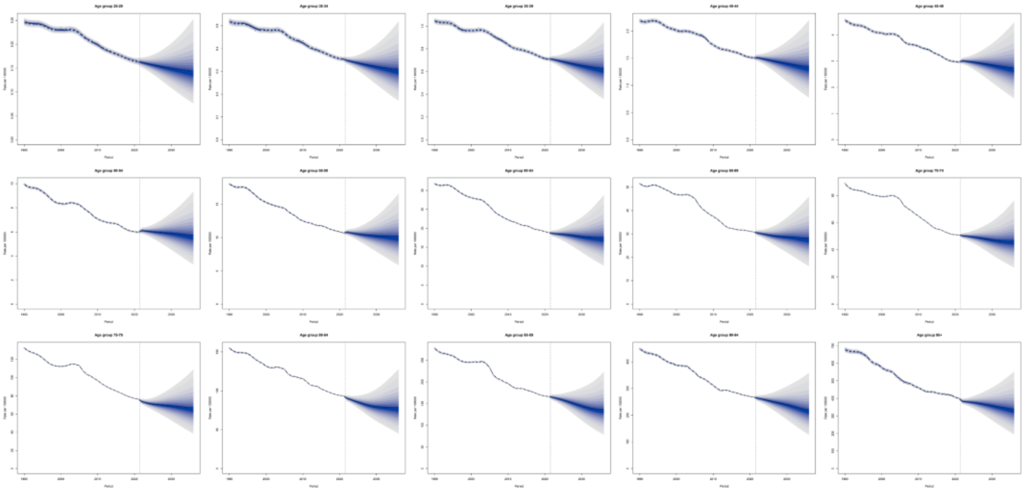

B

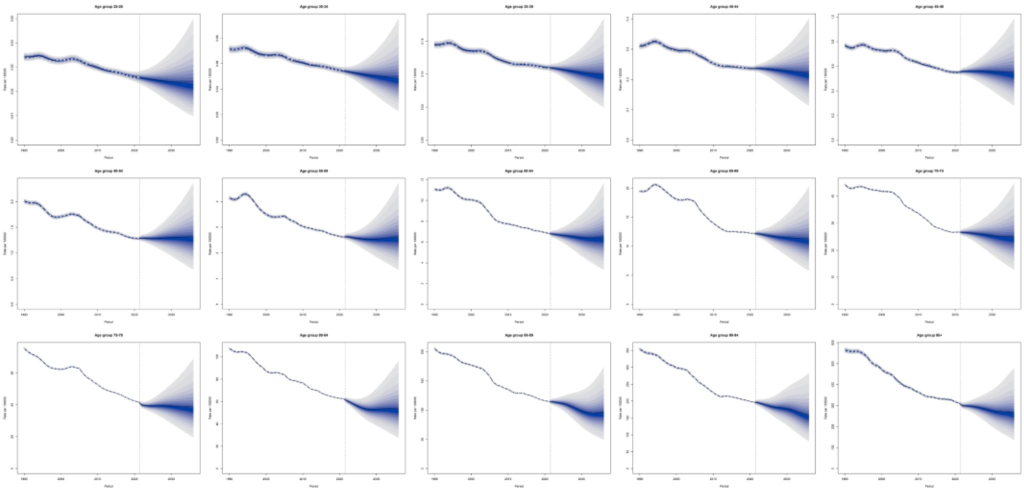

C

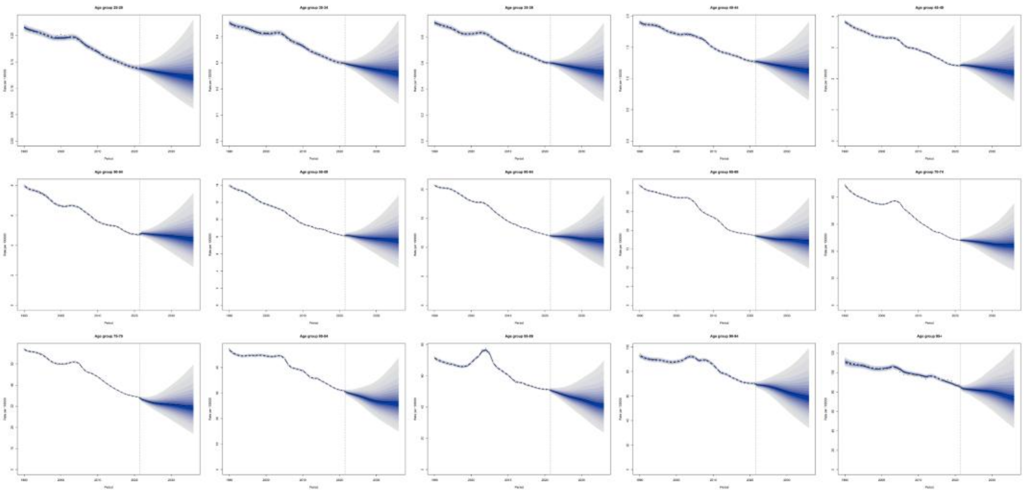

D

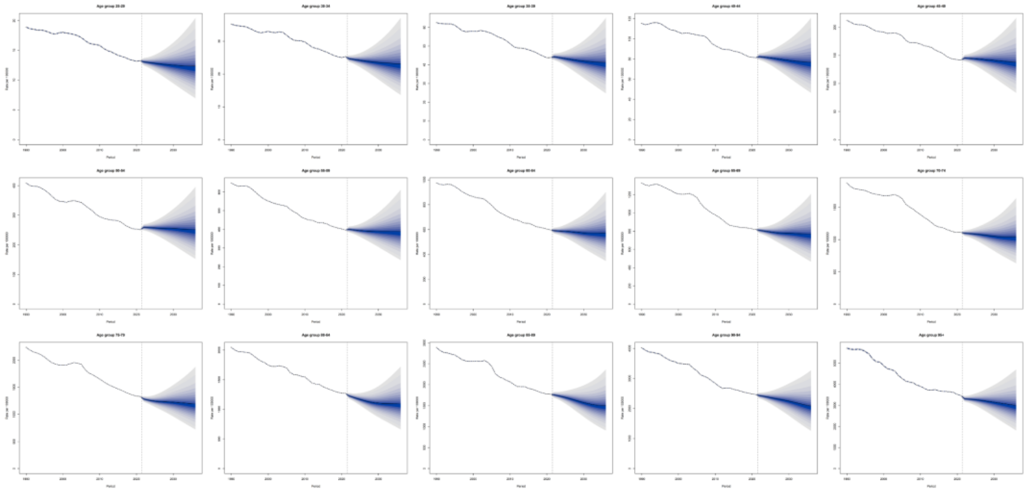

E

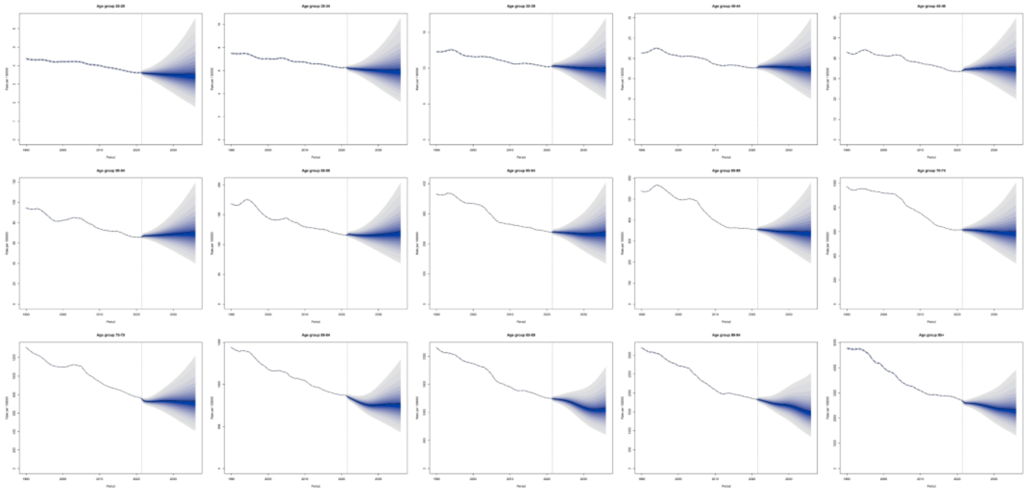

F

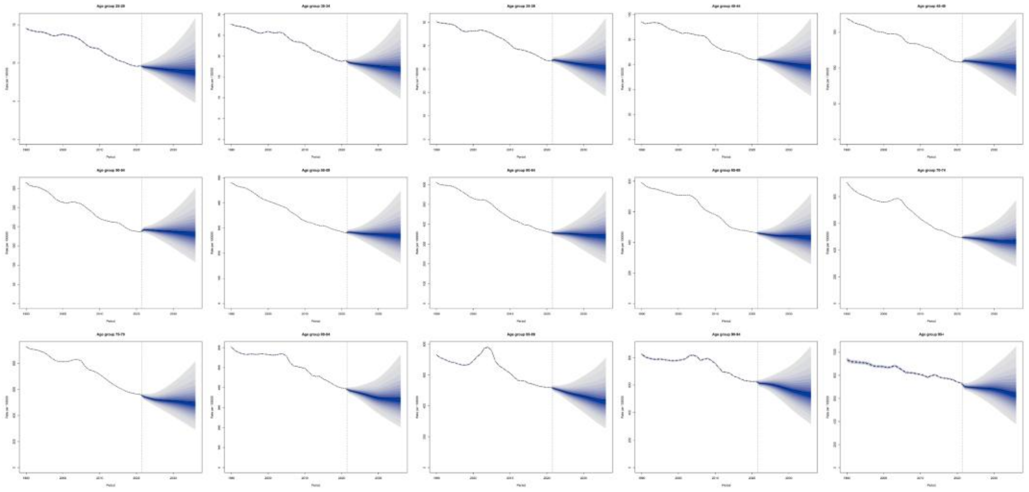

Fig. 3 Trends in age-standardized death rates and DALYs rates of stroke attributable to Kidney Dysfunction from 1990 to 2021, along with forecasts until 2036 across different age groups. The age-standardized death rates and projections for stroke attributable to Kidney Dysfunction are shown for overall Stroke (A), Ischemic stroke (B), and Intracerebral hemorrhage (C). The age-standardized DALYs rates and projections for stroke attributable to Kidney Dysfunction are shown for overall Stroke (D), Ischemic stroke (E), and Intracerebral hemorrhage (F). DALYs stand for Disability-Adjusted Life Years.

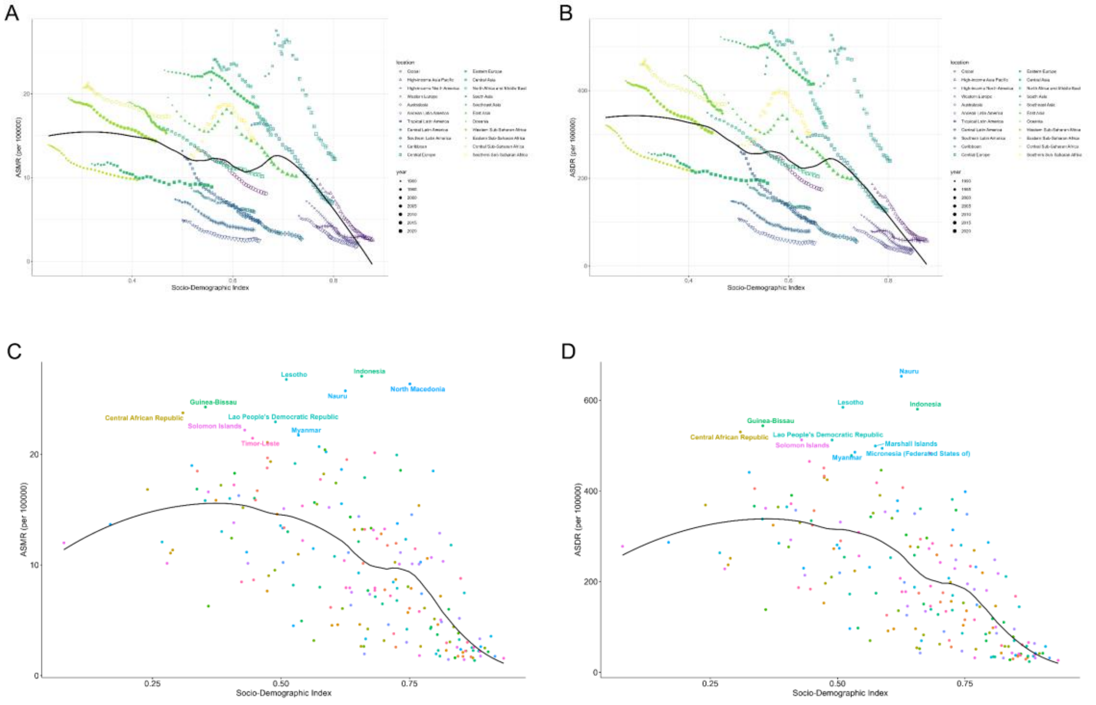

Fig. 4 ASMR and ASDR of stroke attributable to Kidney Dysfunction for 21 regions and 204 countries and territories by SDI. (A) ASMR for 21 regions by SDI from 1990 to 2021. (B) ASDR for 21 regions by SDI from 1990 to 2021. (C) ASMR for 204 countries and territories by SDI in 2021, highlighting the 10 countries and territories with the heaviest burden. (D) ASDR for 204 countries and territories by SDI in 2021, highlighting the 10 countries and territories with the heaviest burden. ASMR Age-Standardized Mortality Rate, ASDR Age-Standardized Disability-Adjusted Life Year Rate, SDI Socio-Demographic Index.

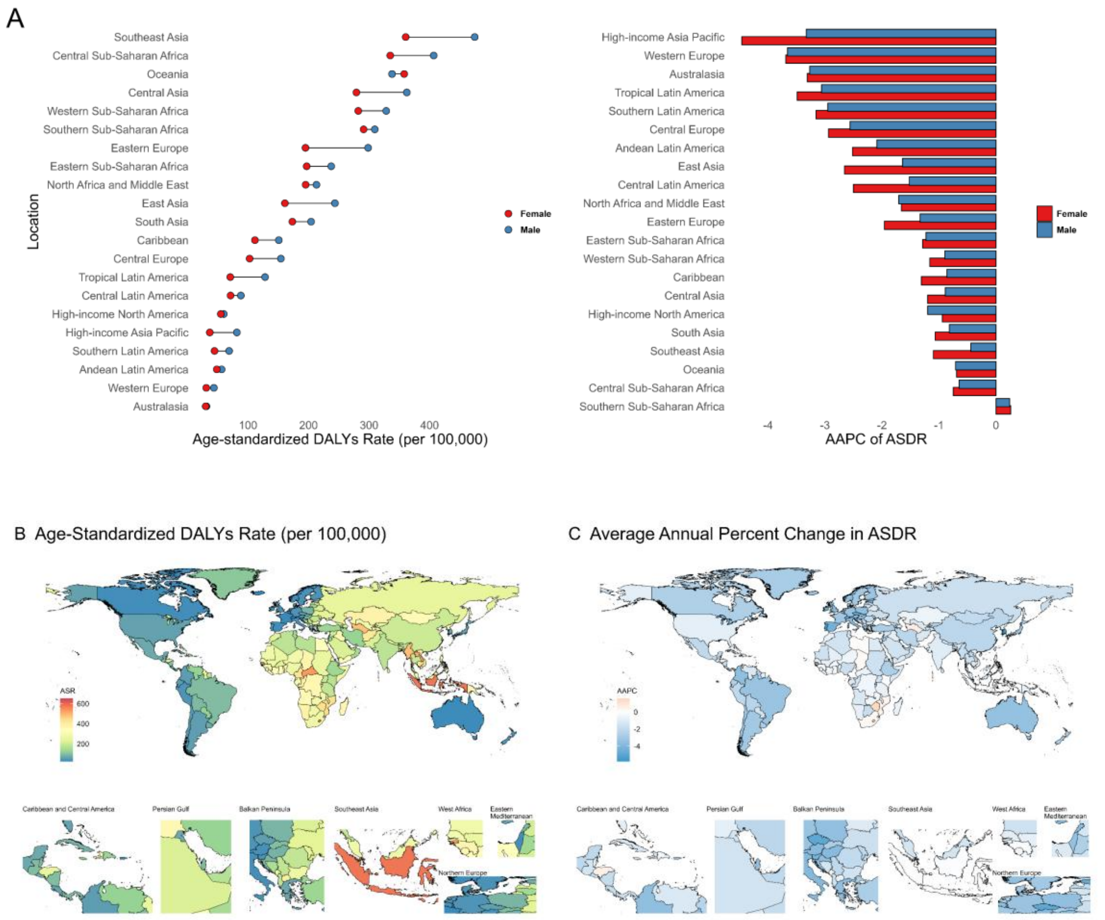

Fig. 5 (A) Age-standardized DALYs rates and their Average Annual Percent Change from 1990 to 2021in stroke attributable to Kidney Dysfunction in 21 regions by sex. (B and C) Age-standardized DALYs rates and their Average Annual Percent Change from 1990 to 2021 in stroke attributable to Kidney Dysfunction by country and territory. DALYs Disability-Adjusted Life Years, AAPC Average Annual Percent Change, ASDR Age-Standardized Disability-Adjusted Life Year Rate.

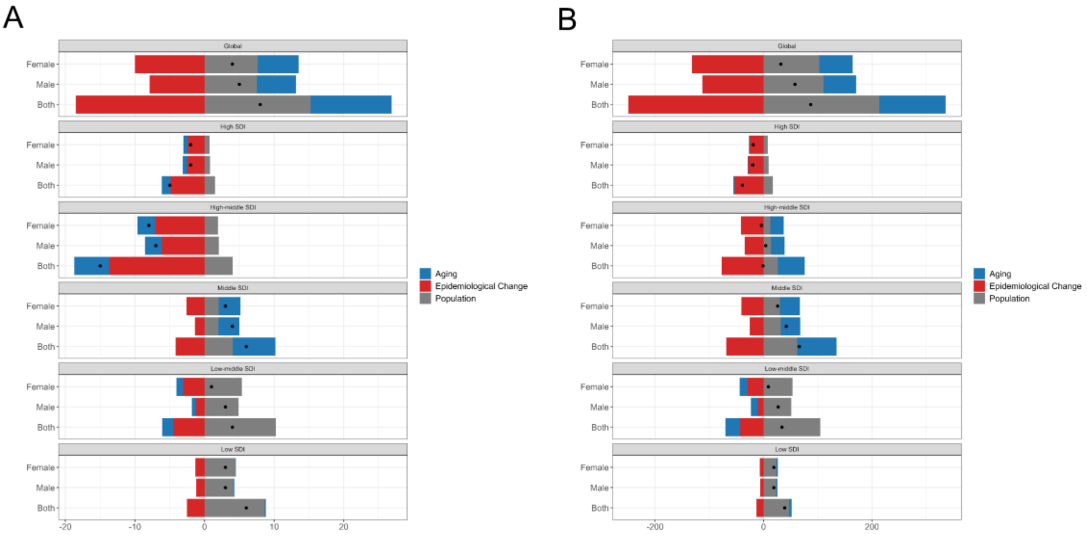

Fig. 6 Changes in age-standardized death rates (A) and DALYs rates (B) in stroke attributable to Kidney Dysfunction according to population-level determinants of aging, population growth, and epidemiological changes from 1990 to 2021 by SDI quintiles. Figures (A) and (B) present data under the conditions of global, high SDI, high-middle SDI, middle SDI, low-middle SDI, and low SDI, respectively. The black dot represents the overall value of change. DALYs Disability-Adjusted Life Years, SDI Socio-Demographic Index.

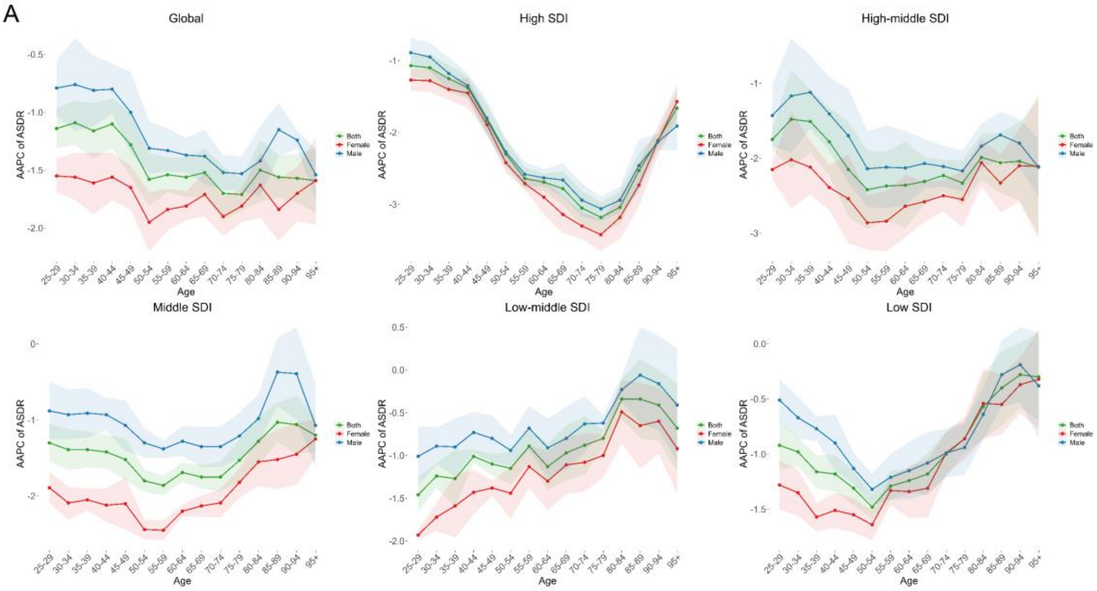

Fig.7 AAPC of age-standardized DALYs rates(A) for stroke attributable to Kidney Dysfunction across different age groups by SDI quintiles from 1990 to 2021. DALYs Disability-Adjusted Life Years, SDI Socio-Demographic Index, AAPC Average Annual Percent Change, ASDR Age-Standardized Disability-Adjusted Life Year Rate.

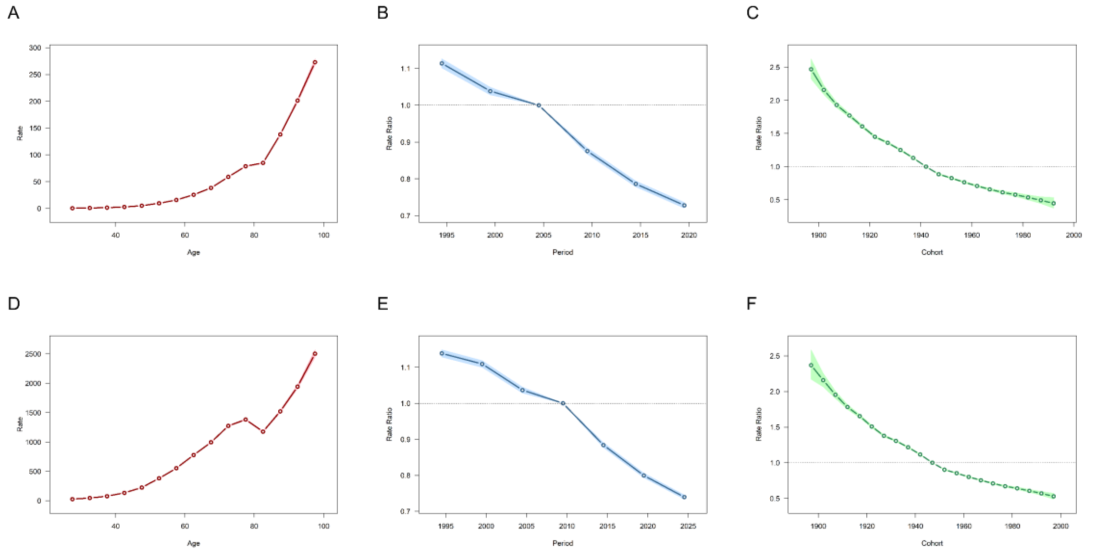

Fig. 8 Analysis of Age, Period, and Birth Cohort Effects on Stroke Attributable to Kidney Dysfunction Globally for ASMR（A, B and C）and ASDR（D, E and F）. ASMR Age-Standardized Mortality Rate, ASDR Age-Standardized Disability-Adjusted Life Year Rate.

Ischemic Stroke

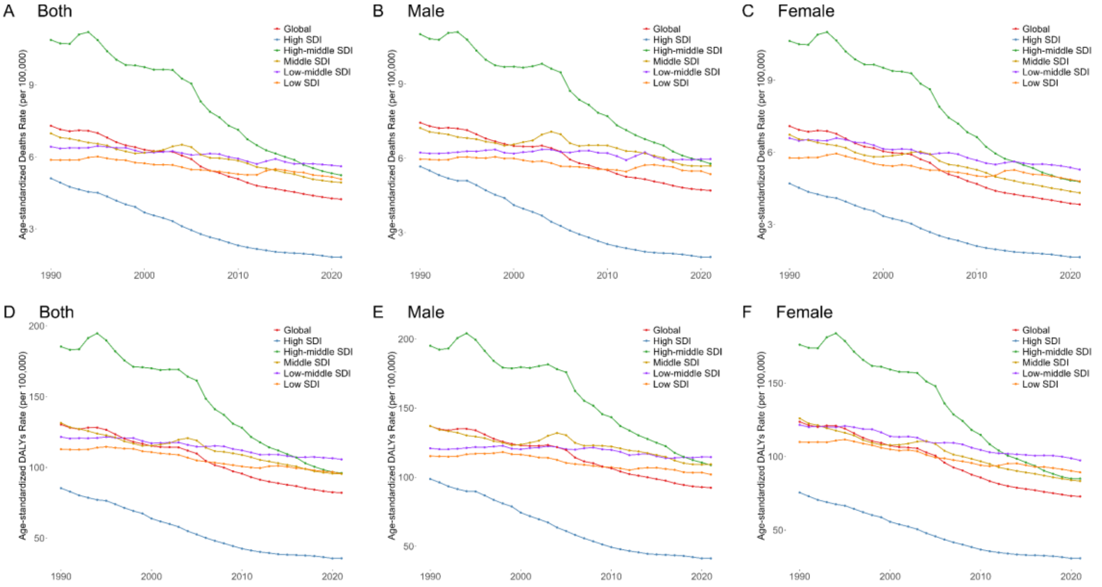

Fig. 9 Age-Standardized Deaths Rate and Age-Standardized DALYs Rate of Ischemic Stroke Attributable to Kidney Dysfunction by SDI Quintiles for Both Sexes (A and D), Male Population (B and E), and Female Population (C and F), 1990–2021. DALYs Disability-Adjusted Life Years, SDI Socio-Demographic Index.

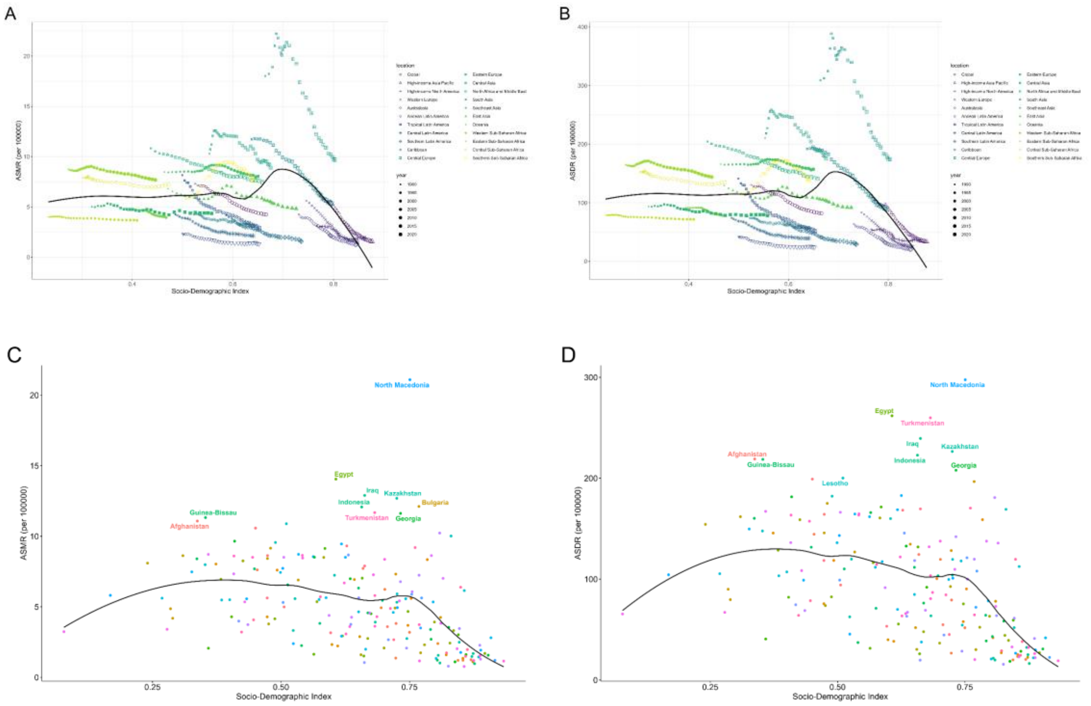

Fig. 10 ASMR and ASDR of Ischemic Stroke attributable to Kidney Dysfunction for 21 regions and 204 countries and territories by SDI. (A) ASMR for 21 regions by SDI from 1990 to 2021. (B) ASDR for 21 regions by SDI from 1990 to 2021. (C) ASMR for 204 countries and territories by SDI in 2021, highlighting the 10 countries and territories with the heaviest burden. (D) ASDR for 204 countries and territories by SDI in 2021, highlighting the 10 countries and territories with the heaviest burden. ASMR age-standardized mortality rate, ASDR Age-Standardized Disability-Adjusted Life Year Rate, SDI Socio-Demographic Index.

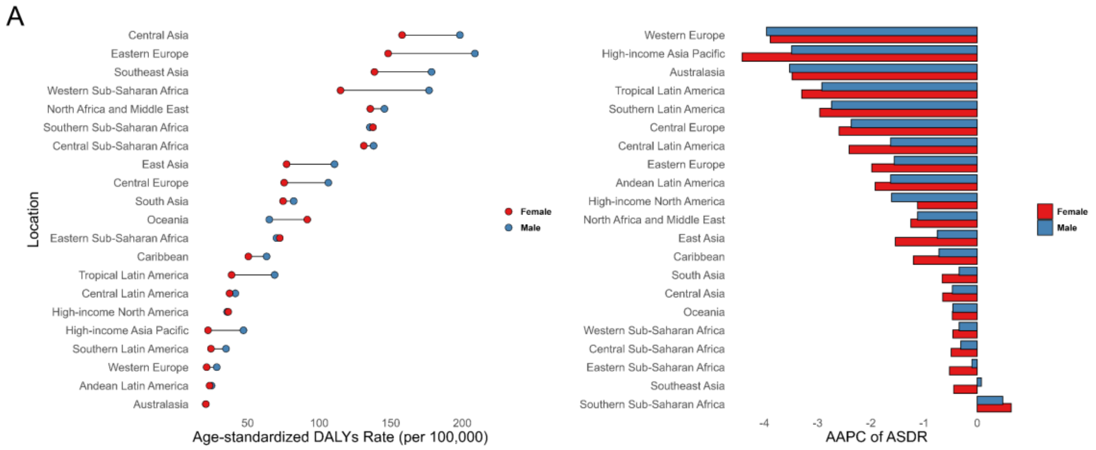

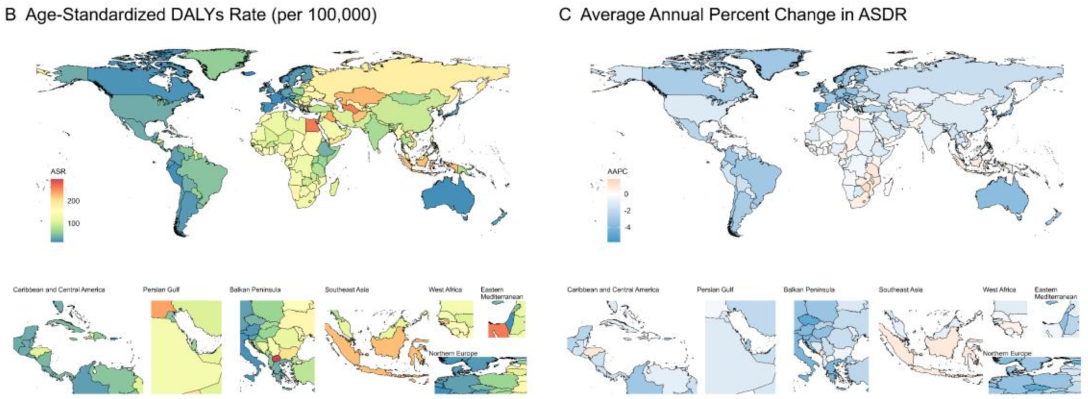

Fig. 11 (A) Age-standardized DALYs rates and their Average Annual Percent Change from 1990 to 2021 in Ischemic Stroke attributable to Kidney Dysfunction in 21 regions by sex. (B and C) Age-standardized DALYs rates and their Average Annual Percent Change from 1990 to 2021 in Ischemic Stroke attributable to Kidney Dysfunction by country and territory. DALYs Disability-Adjusted Life Years, AAPC Average Annual Percent Change, ASDR Age-Standardized Disability-Adjusted Life Year Rate.

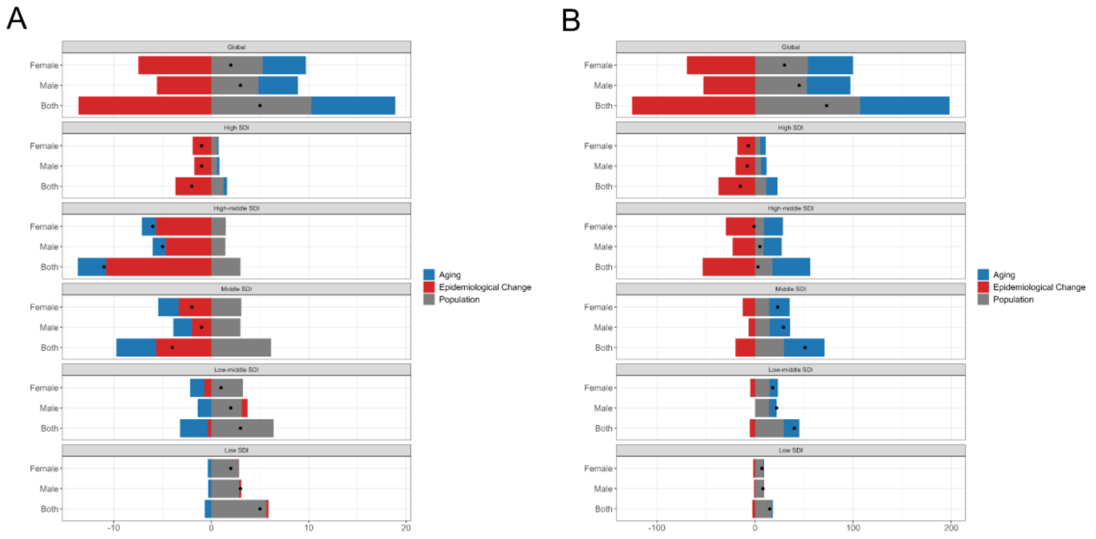

Fig. 12 Changes in age-standardized death rates (A) and DALYs rates (B) in Ischemic Stroke attributable to Kidney Dysfunction according to population-level determinants of aging, population growth, and epidemiological changes from 1990 to 2021 by SDI quintiles. Figures (A) and (B) present data under the conditions of global, high SDI, high-middle SDI, middle SDI, low-middle SDI, and low SDI, respectively. The black dot represents the overall value of change. DALYs Disability-Adjusted Life Years, SDI Socio-Demographic Index.

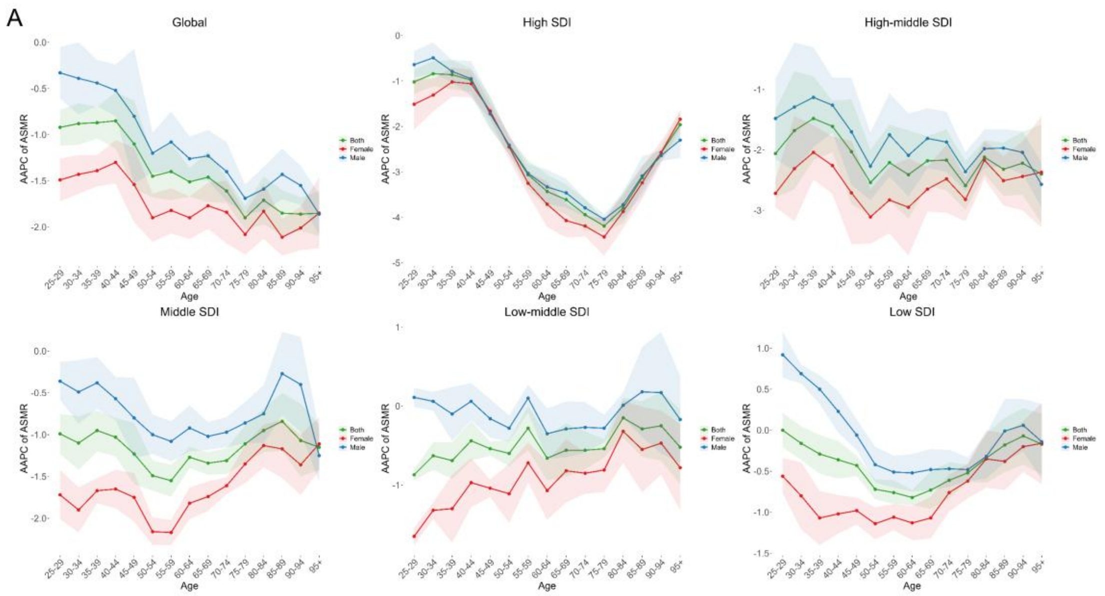

Fig. 13 AAPC of age-standardized death rates(A) for Ischemic Stroke attributable to Kidney Dysfunction across different age groups by SDI quintiles from 1990 to 2021. DALYs Disability-Adjusted Life Years, SDI Socio-Demographic Index, AAPC Average Annual Percent Change, ASMR Age-Standardized Mortality Rate.

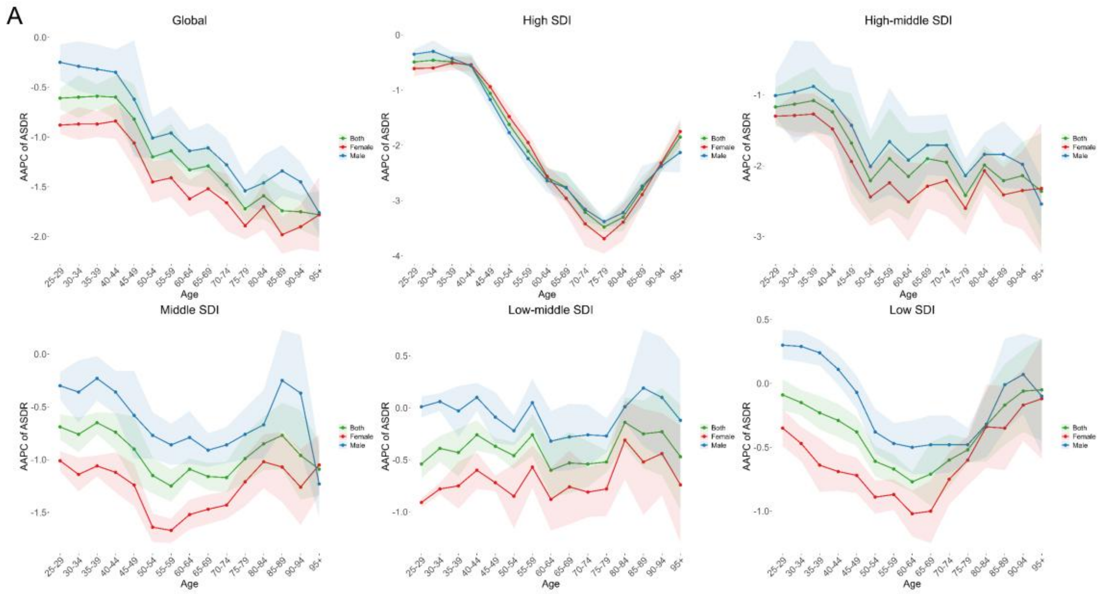

Fig. 14 AAPC of age-standardized DALYs rates(A) for Ischemic Stroke attributable to Kidney Dysfunction across different age groups by SDI quintiles from 1990 to 2021. DALYs Disability-Adjusted Life Years, SDI Socio-Demographic Index, AAPC Average Annual Percent Change, ASDR: age-standardized disability-adjusted life-year rate.

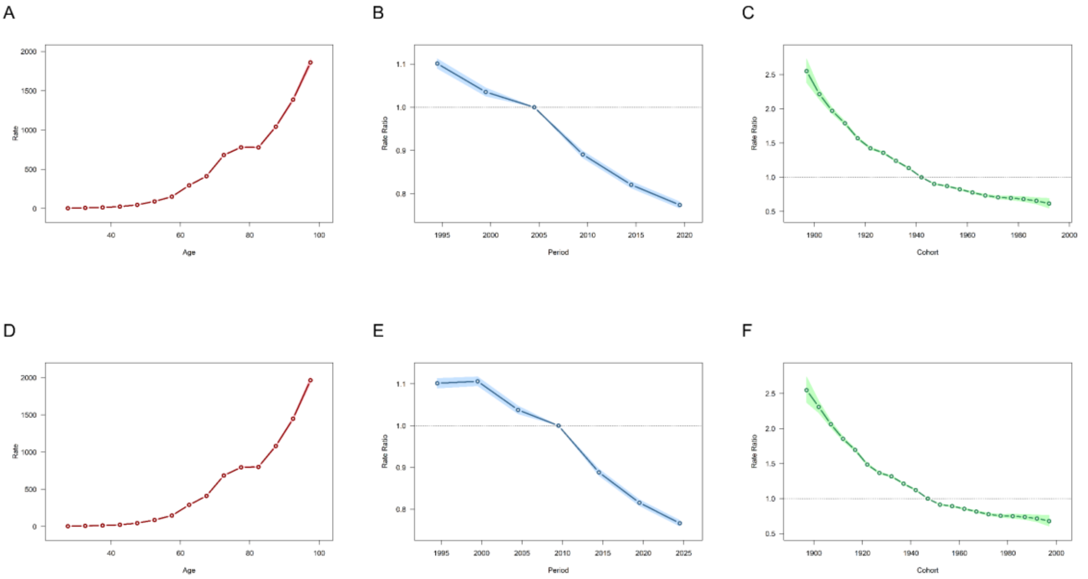

Fig. 15 Analysis of Age, Period, and Birth Cohort Effects on Ischemic Stroke Attributable to Kidney Dysfunction Globally for ASMR（A, B and C）and ASDR（D, E and F）. ASMR Age-Standardized Mortality Rate, ASDR Age-Standardized Disability-Adjusted Life Year Rate.

Intracerebral Hemorrhage

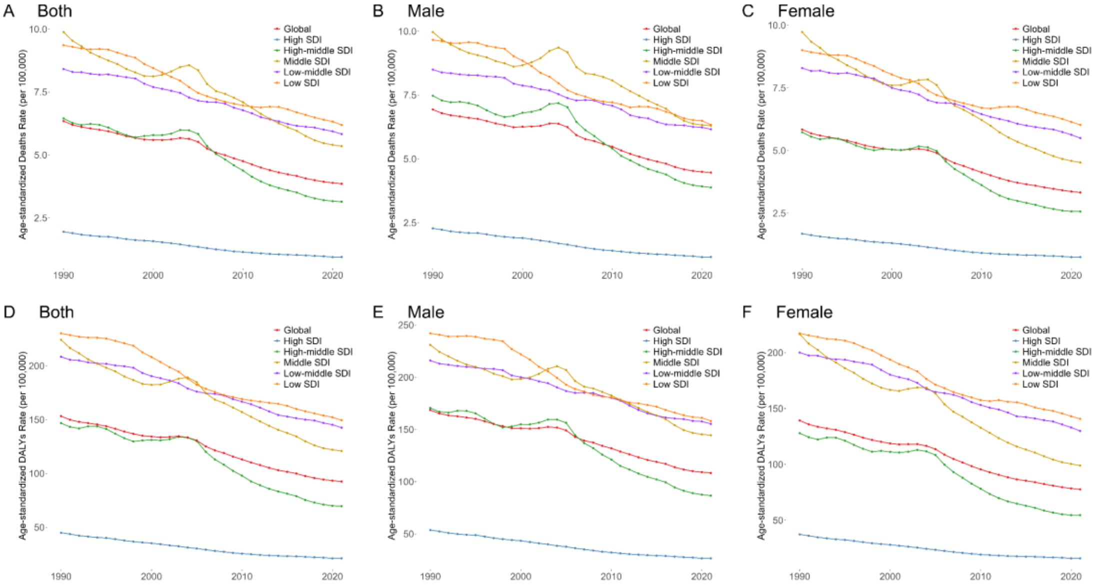

Fig. 16 Age-Standardized Deaths Rate and Age-Standardized DALYs Rate of Intracerebral Hemorrhage Attributable to Kidney Dysfunction by SDI Quintiles for Both Sexes (A and D), Male Population (B and E), and Female Population (C and F), 1990–2021. DALYs Disability-Adjusted Life Years, SDI Socio-Demographic Index.

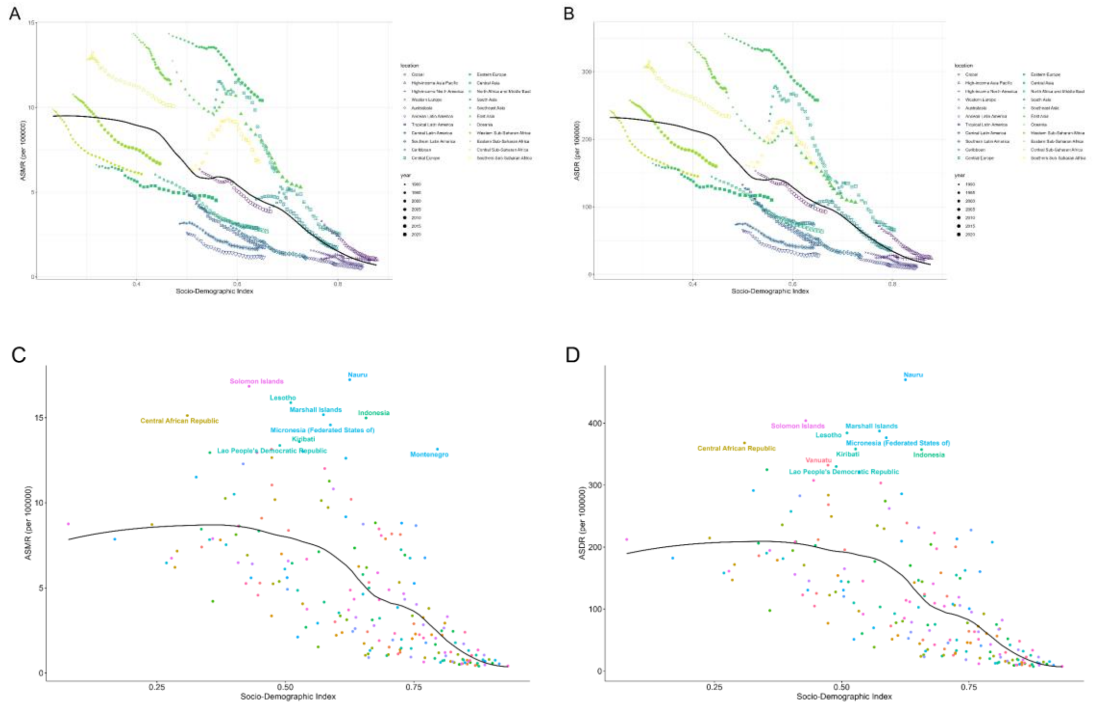

Fig. 17 ASMR and ASDR of Intracerebral Hemorrhage attributable to Kidney Dysfunction for 21 regions and 204 countries and territories by SDI. (A) ASMR for 21 regions by SDI from 1990 to 2021. (B) ASDR for 21 regions by SDI from 1990 to 2021. (C) ASMR for 204 countries and territories by SDI in 2021, highlighting the 10 countries and territories with the heaviest burden. (D) ASDR for 204 countries and territories by SDI in 2021, highlighting the 10 countries and territories with the heaviest burden. ASMR age-standardized mortality rate, ASDR Age-Standardized Disability-Adjusted Life Year Rate; SDI Socio-Demographic Index.

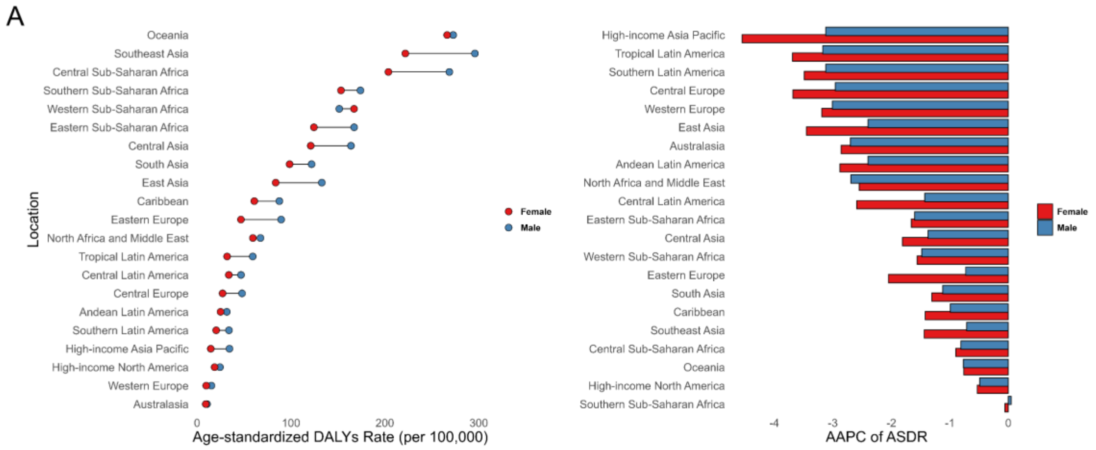

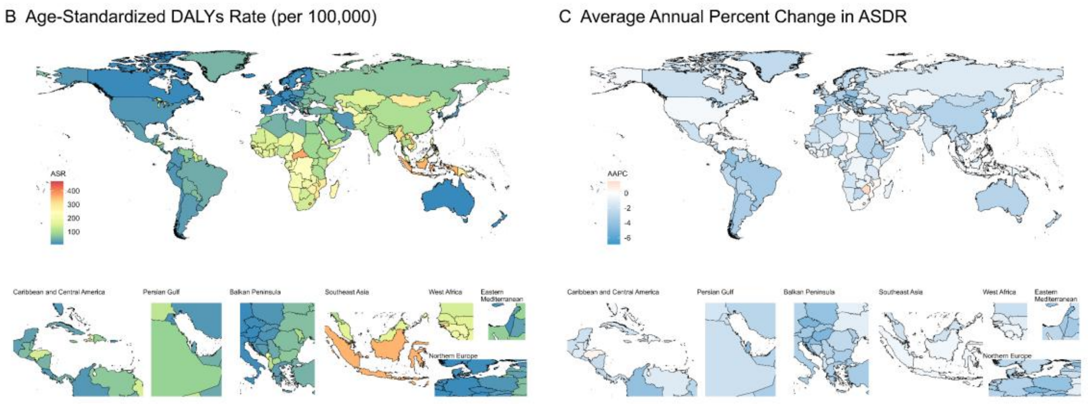

Fig. 18 (A) Age-standardized DALYs rates and their Average Annual Percent Change from 1990 to 2021 in Intracerebral hemorrhage attributable to Kidney Dysfunction in 21 regions by sex. (B and C) Age-standardized DALYs rates and their Average Annual Percent Change from 1990 to 2021 in Intracerebral hemorrhage attributable to Kidney Dysfunction by country and territory. DALYs Disability-Adjusted Life Years, AAPC Average Annual Percent Change.

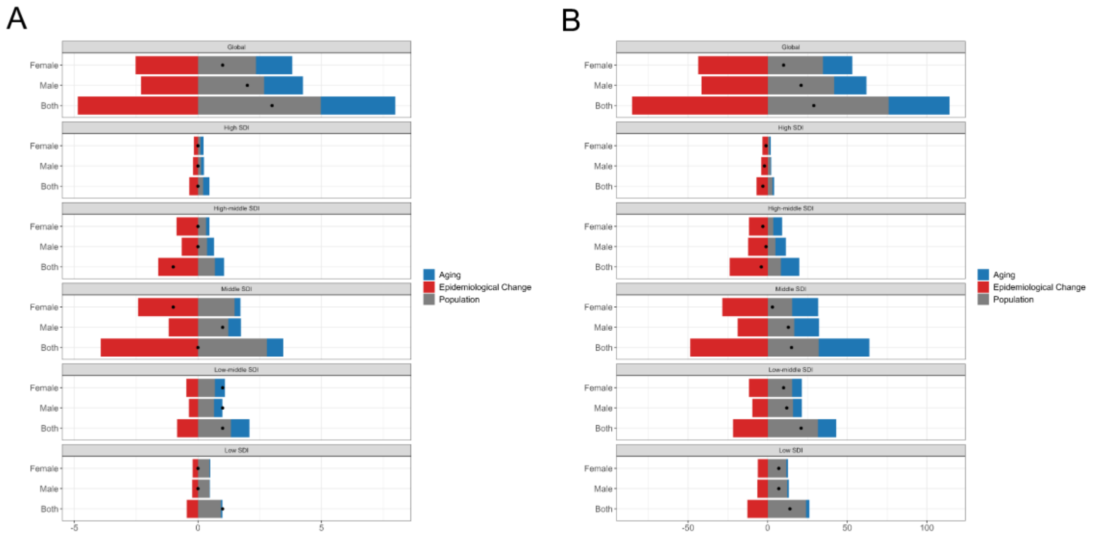

Fig. 19 Changes in age-standardized death rates (A) and DALYs rates (B) in Intracerebral hemorrhage attributable to Kidney Dysfunction according to population-level determinants of aging, population growth, and epidemiological changes from 1990 to 2021 by SDI quintiles. Figures (A) and (B) present data under the conditions of global, high SDI, high-middle SDI, middle SDI, low-middle SDI, and low SDI, respectively. The black dot represents the overall value of change. DALYs Disability-Adjusted Life Years, SDI Socio-Demographic Index.

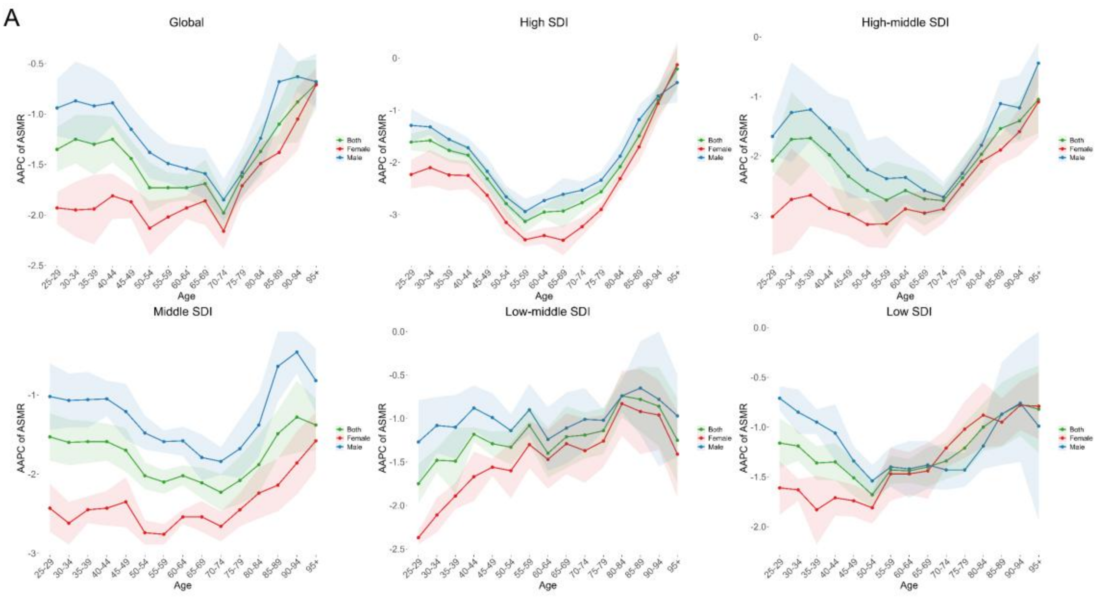

Fig. 20 AAPC of age-standardized death rates(A) for Intracerebral hemorrhage attributable to Kidney Dysfunction across different age groups by SDI quintiles from 1990 to 2021. DALYs Disability-Adjusted Life Years, SDI Socio-Demographic Index, AAPC Average Annual Percent Change, ASMR Age-Standardized Mortality Rate.

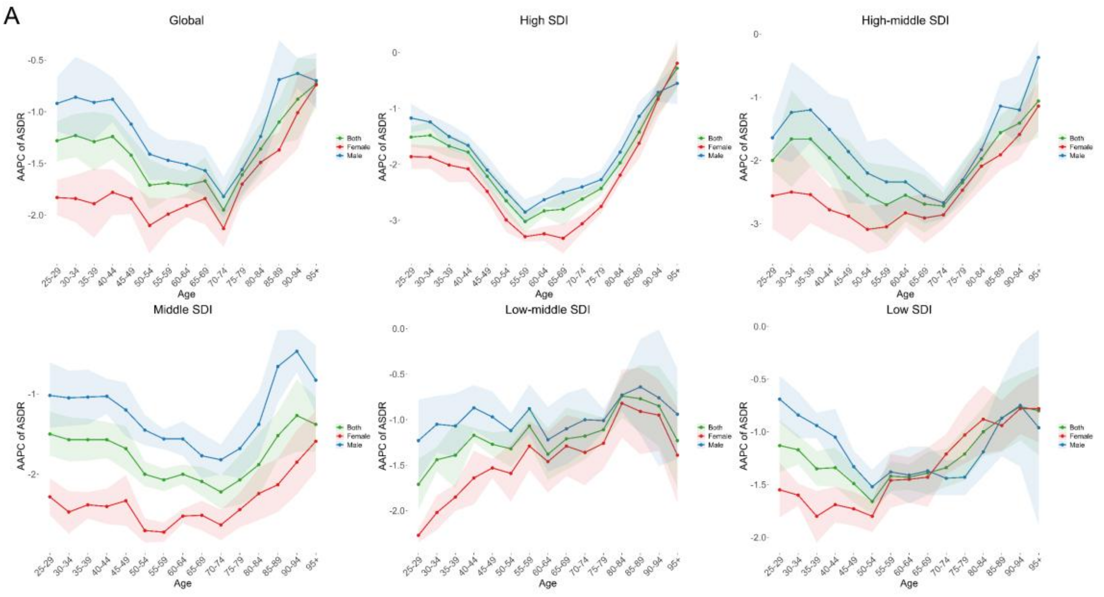

Fig. 21 AAPC of age-standardized DALYs rates(A) for Intracerebral hemorrhage attributable to Kidney Dysfunction across different age groups by SDI quintiles from 1990 to 2021. DALYs Disability-Adjusted Life Years, SDI Socio-Demographic Index, AAPC Average Annual Percent Change, ASDR Age-Standardized Disability-Adjusted Life Year Rate.

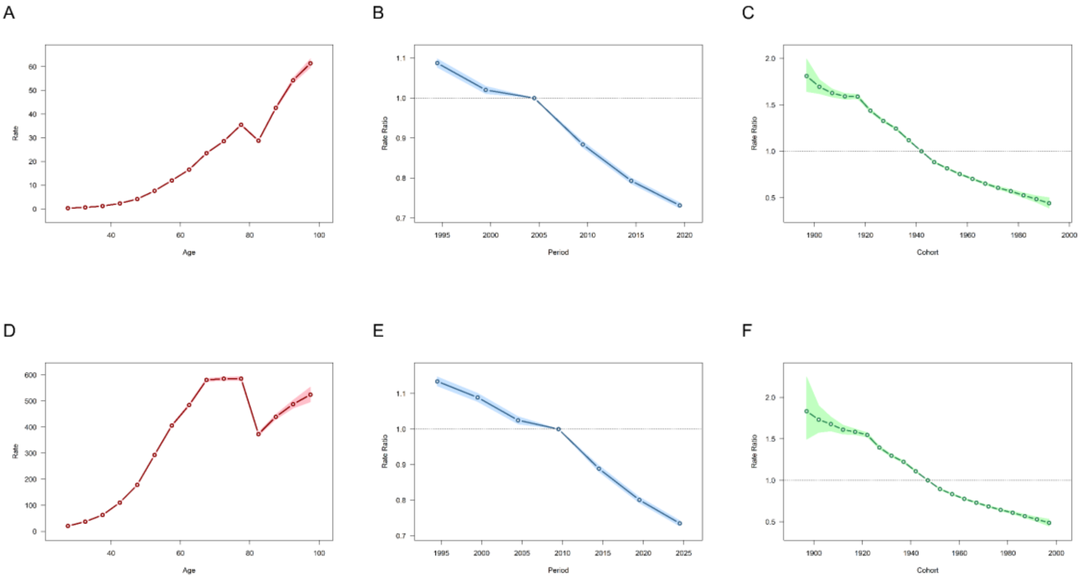

Fig. 22 Analysis of Age, Period, and Birth Cohort Effects on Intracerebral hemorrhage Attributable to Kidney Dysfunction Globally for ASMR（A, B and C） and ASDR（D, E and F）. ASMR Age-Standardized Mortality Rate, ASDR Age-Standardized Disability-Adjusted Life Year Rate.

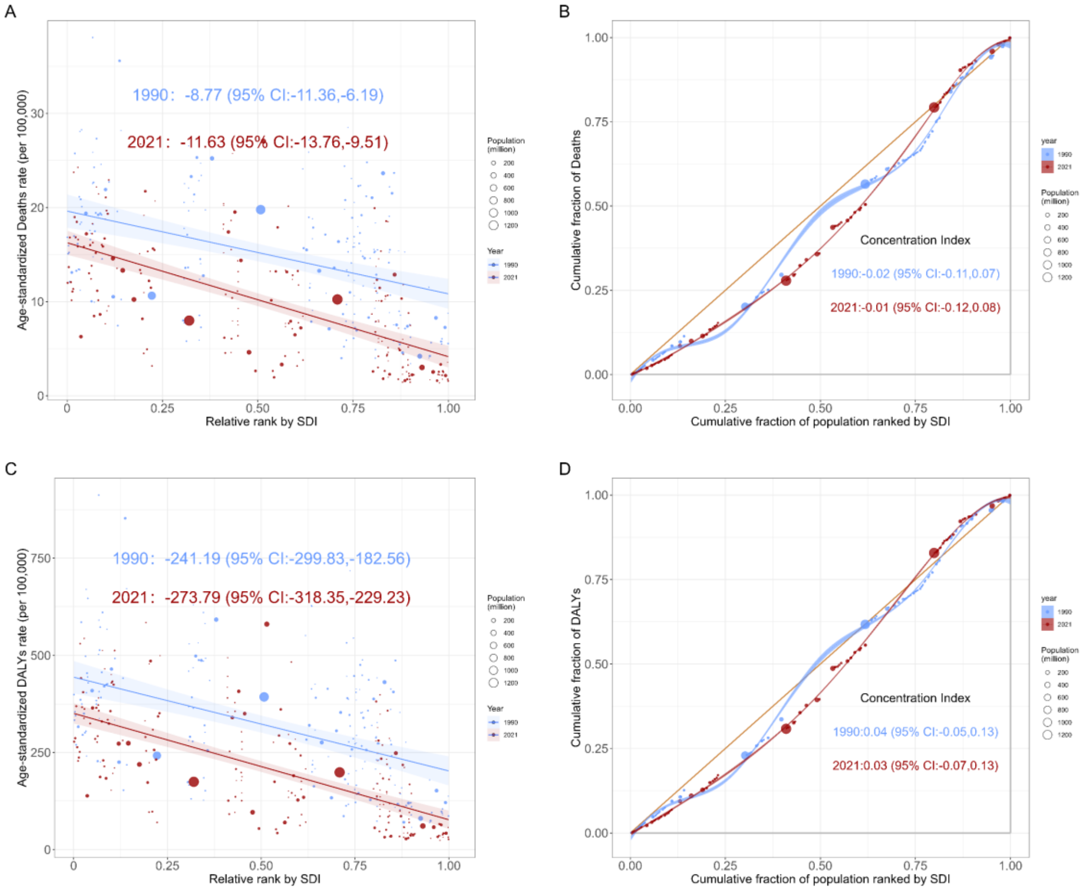

Fig. 23 Slope Index of Inequality curves and Concentration Index curves for deaths and DALYs due to Stroke attributable to Kidney Dysfunction, 1990 and 2021. **(A)** Slope Index of Inequality curves for the age-standardized death rate of Stroke. **(B)** Concentration Index curves for Deaths of Stroke. **(C)** Slope Index of Inequality curves for age-standardized DALYs rate of Stroke. **(D)** Concentration Index curves for DALYs of Stroke. DALYs Disability-Adjusted Life Years.

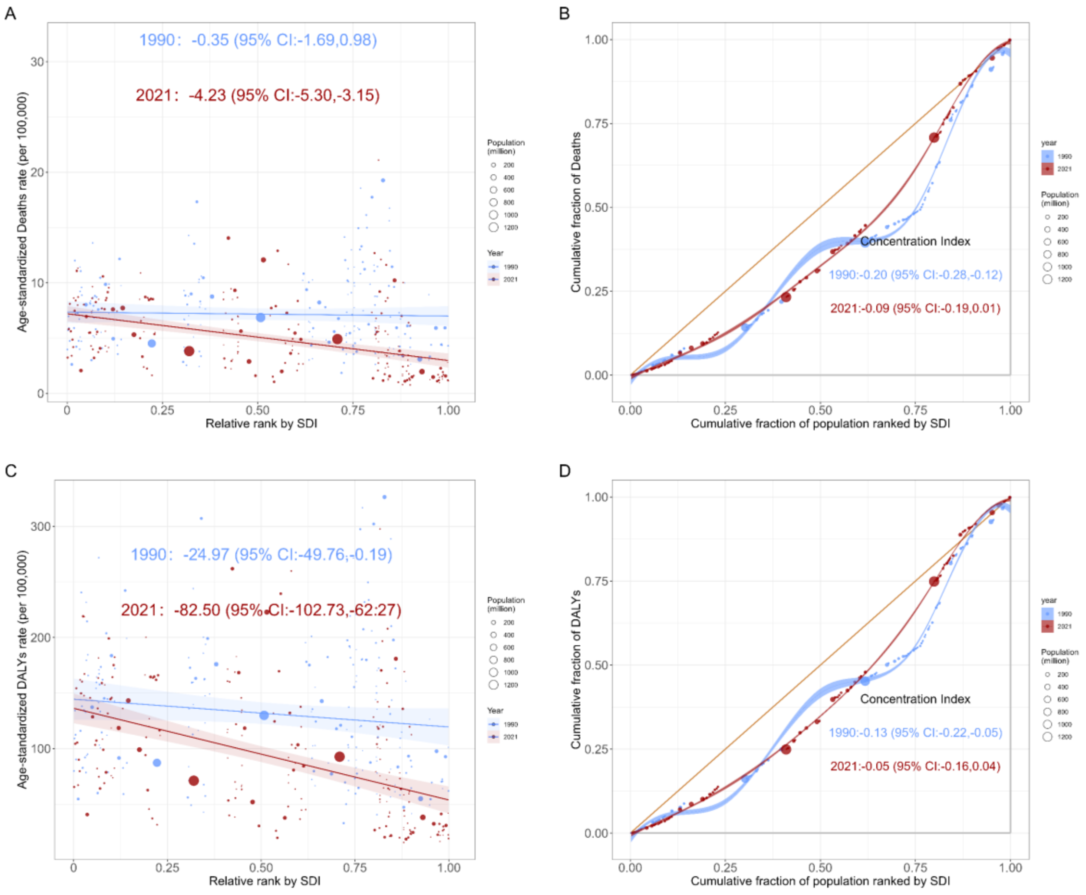

Fig. 24 Slope Index of Inequality curves and Concentration Index curves for deaths and DALYs due to ischemic stroke attributable to Kidney Dysfunction, 1990 and 2021. **(A)** Slope Index of Inequality curves for the age-standardized death rate of ischemic stroke. **(B)** Concentration Index curves for Deaths of ischemic stroke. **(C)** Slope Index of Inequality curves for age-standardized DALYs rate of ischemic stroke. **(D)** Concentration Index curves for DALYs of ischemic stroke. DALYs Disability-Adjusted Life Years.

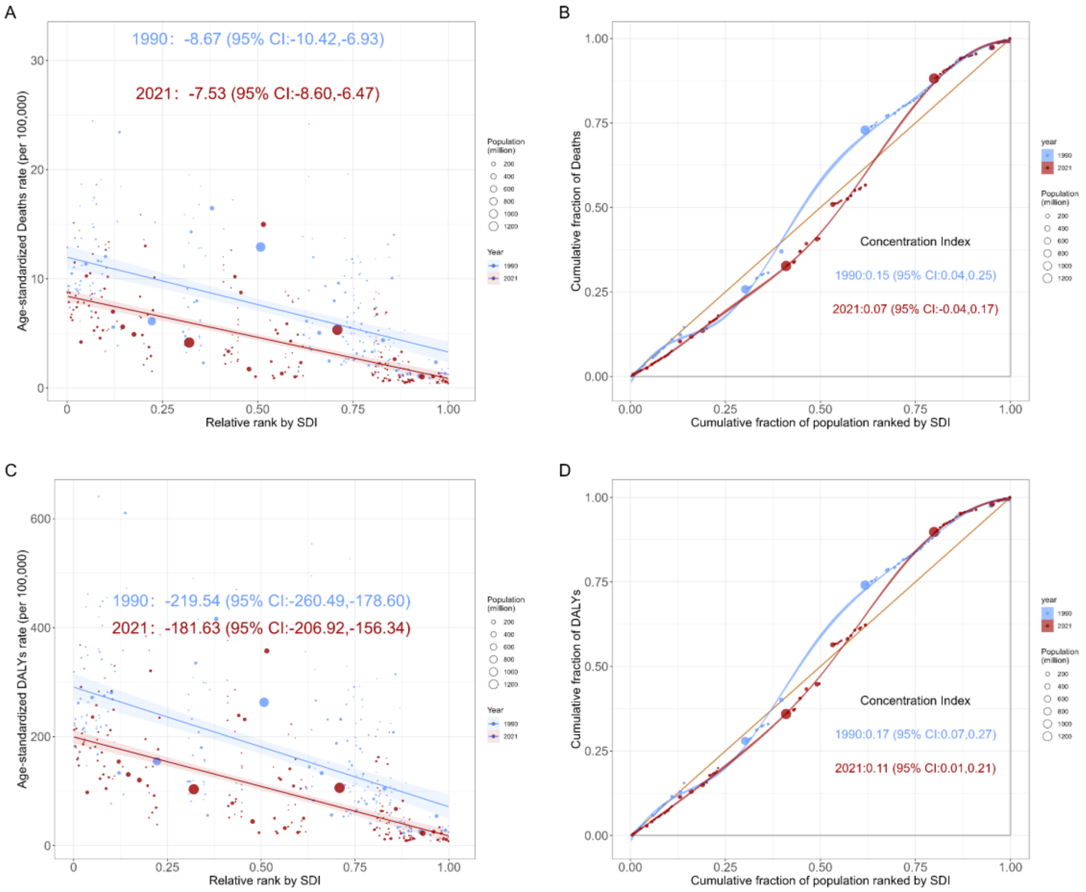

Fig. 25 Slope Index of Inequality curves and Concentration Index curves for deaths and DALYs due to Intracerebral hemorrhage attributable to Kidney Dysfunction, 1990 and 2021. **(A)** Slope Index of Inequality curves for the age-standardized death rate of Intracerebral hemorrhage. **(B)** Concentration Index curves for Deaths of Intracerebral hemorrhage. **(C)** Slope Index of Inequality curves for age-standardized DALYs rate of Intracerebral hemorrhage. **(D)** Concentration Index curves for DALYs of Intracerebral hemorrhage. DALYs Disability-Adjusted Life Years.
